# Disruption of mitochondrial quality control genes promotes caspase-resistant cell survival following apoptotic stimuli

**DOI:** 10.1016/j.jbc.2022.101835

**Published:** 2022-03-16

**Authors:** Yulia Kushnareva, Vivian Moraes, Julian Suess, Bjoern Peters, Donald D. Newmeyer, Tomomi Kuwana

**Affiliations:** 1 Division of Immune Regulation, La Jolla Institute for Immunology, La Jolla, California, USA; 2 Department of Biochemical Pharmacology, University of Konstanz, Konstanz, Germany

**Keywords:** mitochondrial quality control, mitochondrial dynamics, mitophagy, mitochondrial heterogeneity, apoptosis, mitochondrial outer membrane permeabilization, siRNA screen, oncogenesis, APAF-1, apoptotic protease-activating factor-1, BAK, Bcl-2 homologous antagonist/killer, BAX, BCL2-associated X, apoptosis regulator, BCL2, B-cell lymphoma 2, BH3, Bcl-2 homology domain 3, BNIP3, Bcl-2/adenovirus E1B 19-kDa interacting protein 3, DMEM, Dulbecco's modified Eagle's medium, DRP1, dynamin-related protein 1, ER, endoplasmic reticulum, FBS, fetal bovine serum, MEF, mouse embryonic fibroblast, MFN, mitofusin, MOM, mitochondrial outer membrane, MOMP, mitochondrial outer membrane permeabilization, MQC, mitochondrial quality control, OPA1, optic atrophy 1, Q-VD, quinoline-Val-Asp-difluorophenoxymethylketone, sgRNA, single-guide RNA, SMAC, second mitochondria-derived activator of caspase

## Abstract

In cells undergoing cell-intrinsic apoptosis, mitochondrial outer membrane permeabilization (MOMP) typically marks an irreversible step in the cell death process. However, in some cases, a subpopulation of treated cells can exhibit a sublethal response, termed “minority MOMP.” In this phenomenon, the affected cells survive, despite a low level of caspase activation and subsequent limited activation of the endonuclease caspase-activated DNase (DNA fragmentation factor subunit beta). Consequently, these cells can experience DNA damage, increasing the probability of oncogenesis. However, little is known about the minority MOMP response. To discover genes that affect the MOMP response in individual cells, we conducted an imaging-based phenotypic siRNA screen. We identified multiple candidate genes whose downregulation increased the heterogeneity of MOMP within single cells, among which were genes related to mitochondrial dynamics and mitophagy that participate in the mitochondrial quality control (MQC) system. Furthermore, to test the hypothesis that functional MQC is important for reducing the frequency of minority MOMP, we developed an assay to measure the clonogenic survival of caspase-engaged cells. We found that cells deficient in various MQC genes were indeed prone to aberrant post-MOMP survival. Our data highlight the important role of proteins involved in mitochondrial dynamics and mitophagy in preventing apoptotic dysregulation and oncogenesis.

Apoptosis is a ubiquitous cellular self-elimination process that is critical for the homeostasis of various cell populations. Dysregulated apoptosis is integral to cancer progression and contributes to multiple diseases, including immune and neurodegenerative disorders. Many cancer therapies rely on the enhanced apoptotic death of tumor cells. Apoptosis frequently involves a “cell-intrinsic” pathway involving mitochondria. The central event in mitochondria-dependent apoptosis is mitochondrial outer membrane permeabilization (MOMP), which is induced by B-cell lymphoma 2 (BCL2)–associated X, apoptosis regulator (BAX) and Bcl-2 homologous antagonist/killer (BAK), the key proapoptotic BCL-2 family proteins (1, 2). BAK is constitutively located on the mitochondrial outer membrane (MOM), whereas BAX is mostly soluble in the cytoplasm. When cells receive an apoptotic stress signal, molecules belonging to a subset of the BCL-2 family termed “Bcl-2 homology domain 3 (BH3)–only proteins” activate BAX and BAK. Consequently, BAX translocates to the MOM, and both BAX and BAK integrate into the outer membrane and trigger MOMP by inducing the formation of large membrane pores (3, 4, 5, 6, 7). In concert with these BCL-2 family members, additional MOM proteins facilitate BAX-induced pore formation (4). Antiapoptotic BCL-2 family members (including BCL-2, BCL-extra large, and myeloid cell leukemia-1) inhibit MOMP by sequestering the BH3-only proteins and by antagonizing the pore-forming activity of BAX and BAK.

The apoptotic pores in the MOM allow proteins normally residing in the mitochondrial intermembrane space to escape into the cytoplasm. Certain of these proteins are apoptogenic, including cytochrome *c*, second mitochondria-derived activator of caspase (SMAC)/direct inhibitor of apoptosis-binding protein with low pI, and OMI/high-temperature requirement factor A1. These proteins induce apoptotic protease-activating factor-1 (APAF-1)–dependent activation of caspase-9 and the effector caspases (most prominently caspase-3), which cleave certain protein substrates that actively promote cell destruction and engulfment (8, 9, 10). Even when caspase activity is blocked, MOMP *per se* usually leads to cell death, as it compromises energy metabolism in the permeabilized mitochondria (11, 12, 13). Although mitochondrial membrane potential and cellular ATP are maintained for some time after MOMP, cells treated with caspase inhibitors typically exhibit a gradual decline in mitochondrial respiration and a complete loss of clonogenic survival over the course of 2 or 3 days (12).

Recent reports describe a phenomenon, termed “minority MOMP,” in which cells exposed to relatively weak cell stressors can evade MOMP-dependent death (14, 15, 16). In these atypical cells, only a fraction of the cell’s mitochondria undergoes MOMP. As a result of minority MOMP, small amounts of cytochrome *c* and SMAC are released into the cytosol, and downstream caspases become activated at sublethal levels. This low-level caspase activation leads to a correspondingly limited activation of the apoptotic caspase-activated DNase (DNA fragmentation factor subunit beta) endonuclease, which enters the cell nucleus and produces a degree of DNA damage. The surviving cells exhibit genome instability and an increased propensity to become oncogenic (17).

The mechanisms controlling the uniformity of MOMP in cells, and hence the frequency of minority MOMP, are unclear. To discover genes that affect MOMP, we conducted a focused high-content siRNA screen that analyzed 1318 genes that had been annotated in databases as having some relationship to mitochondria. A novel aspect of our screen was the identification of genes whose silencing produced a heterogeneous MOMP phenotype in which some mitochondria within the cell were permeabilized, whereas others remained intact. Our screen yielded functionally diverse candidate genes, including a significant group of genes associated with mitophagy, autophagy, and mitochondrial dynamics, the processes constituting the system of mitochondrial quality control (MQC) (18, 19). We hypothesized that an increased heterogeneity of MOMP observed in MQC-compromised cells could increase the frequency of minority MOMP (17). If so, downregulation of MQC-related genes would tend to allow cells to survive stresses that produce a degree of caspase activation, as a result of limited MOMP. To test this hypothesis, we developed a single-cell assay to measure the long-term clonogenic cell survival of cells that had exhibited a measurable degree of caspase activation, in response to treatment with a BH3-mimetic drug. Our results confirmed that deficiencies in various MQC proteins enhanced the survival of caspase-engaged cells. We conclude that mitochondrial dynamics and mitophagy play a critical role in limiting the frequency of minority MOMP. Thus, proper functioning of the MQC system can be important to prevent oncogenesis that results from an incomplete execution of the mitochondrial apoptosis program.

## Results and discussion

### Screening strategy and assay development

To identify novel cell-intrinsic regulators of MOMP, we designed a high-content image-based siRNA screen. As we were primarily interested in mitochondrial function, we carried out a focused siRNA screen targeting genes with annotated relationships to mitochondria (Table S1). Our strategy took advantage of a HeLa cell line expressing two fluorescent reporter proteins (described previously by Llambi *et al.* (20)) that enabled us to simultaneously interrogate both BAX activation and MOMP. In living cells, Venus-BAX is cytoplasmic (diffuse), and OMI-mCherry is localized to mitochondria. When apoptosis is induced, Venus-BAX is translocated to mitochondria and OMI-mCherry is released into the cytoplasm and degraded. Normally, once apoptotic BAX translocation is initiated in a given cell, mitochondrial intermembrane space proteins (including cytochrome *c* or OMI) are released in a synchronous and rapid fashion (14, 20, 24). The release of proteins from the mitochondrial interior can only be restricted under special circumstances, for example, cristae junction remodeling by overexpression of a mutant form of the optic atrophy 1 (OPA1) protein (25) or MOMP inhibition by a recently described endolysosome-linked mechanism (26). However, in some cells, the release of intermembrane proteins is not an all-or-none event, and mitochondria within the same cell display heterogeneity in MOMP response (16, 17). We hypothesized that downregulation of certain genes could increase MOMP heterogeneity and promote post-MOMP cell survival, even in caspase-proficient cells.

**Figure 1:**
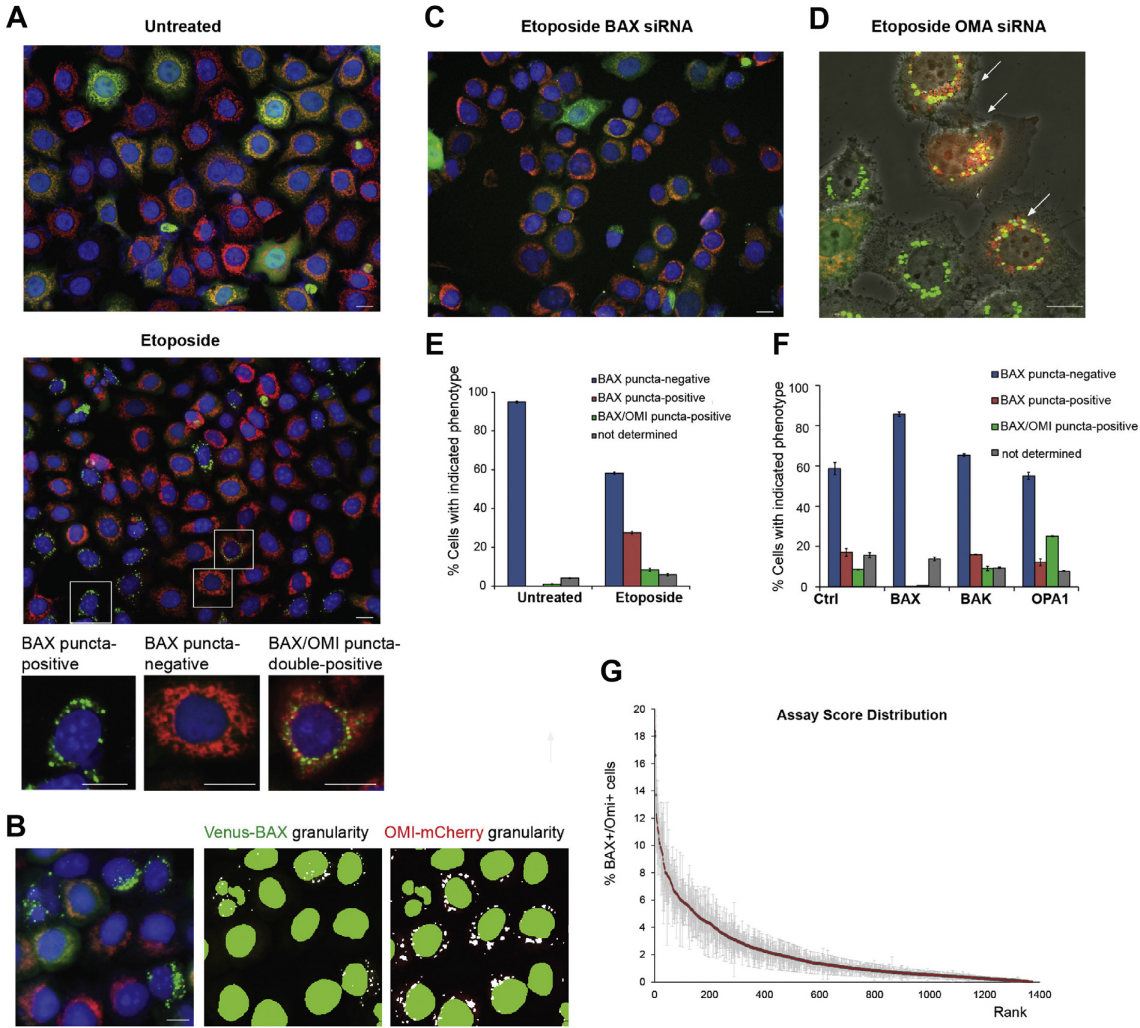
**Imaging-based screening assay for regulators of apoptotic MOMP response.***A*, representative epifluorescence microscopy of untreated and etoposide-treated cells expressing Venus-BAX (*green*; YFP channel) and OMI-mCherry (*red*; Texas Red channel). *Bottom panel* illustrates enlarged cells with indicated phenotypes. Nuclei were stained with Hoechst-33342 (*blue*; DAPI channel). *B*, identification of Venus-BAX and OMI-mCherry puncta using MetaXpress granularity application module. A representative enlarged image of etoposide-treated cells (*left panel*) and corresponding image segmentations. The granularity module identifies Venus-BAX (*middle panel*) and OMI-mCherry puncta. Note that cells with BAX “granules” do not contain OMI “granules” and *vice versa*. Nuclear segmentation settings correctly identify fragmented (apoptotic) and normal size nuclei. *C*, BAX siRNA inhibits Venus-BAX puncta formation and OMI-mCherry release in etoposide-treated cells. *D*, a confocal image of live Venus-BAX/OMI-mCherry cells transfected OMA1 siRNA. *Arrows* indicate cells with heterogeneous MOMP. The scale bars in (*A–D*) represent 20 μm. *E* and *F*, examples of phenotype quantification using granularity application module and high-throughput microscopy. *E*, quantification of indicated phenotypes in untreated and etoposide-treated cells transfected with a nontargeting (*control*) siRNA; “nondetermined” phenotype corresponds to a small fraction of nonfluorescent cells. *F*, effects of indicated siRNAs on the phenotype distribution. Data are mean ± SEM (n = 3 replicate wells). *G*, sorted assay scores for the primary screen siRNA set in triplicate plates. Numbers indicate the percentage of cells positive for both Venus-BAX and OMI-mCherry foci. Values shown are mean and SEM, n = 3. Hits were identified from the top 10% tail of the BAX/OMI score. BAX, BCL2-associated X, apoptosis regulator; DAPI, 4′,6-diamidino-2-phenylindole; MOMP, mitochondrial outer membrane permeabilization.

To begin to test our hypothesis, we analyzed punctate mitochondrial distribution of Venus-BAX and OMI-mCherry in cells treated with the apoptosis inducer, etoposide. The caspase inhibitor quinoline-Val-Asp-difluorophenoxymethylketone (Q-VD) was included in these experiments to decrease premature cell loss and detachment. Previous studies have demonstrated that caspase inhibition does not affect the kinetics of the release of intermembrane space proteins (12, 24, 27). We configured our automated image analysis to quantify three cellular phenotypes: (1) apoptotic cells with mitochondria that have undergone MOMP-containing translocated BAX and lacking intramitochondrial OMI (BAX puncta positive), (2) nonapoptotic cells with intact mitochondria lacking BAX and containing OMI (BAX puncta negative), and (3) atypical cells with mitochondria containing BAX but which have not released OMI (BAX/OMI puncta double-positive cells) (Fig. 1. *A* and *B*). Untreated (no etoposide) cells transfected with control siRNA were nonapoptotic and showed normal mitochondrial morphology (Fig. 1, *A* and *E*) indicating that our optimized transfection conditions produced minimal toxicity. As expected, most etoposide-treated cells fell into either of the first two categories, whereas BAX/OMI double-positive cells were minimally present (Fig. 1, *A* and *E*). In assay validation experiments, we tested the effect of OPA1 siRNA. OPA1 mediates mitochondrial fusion in conjunction with its cleavage by the mitochondrial protease OMA1 (28, 29, 30) and has multiple roles in apoptotic signaling (22, 25, 31, 32, 33). Knockdown of OPA1 protein increased the number of BAX/OMI double-positive cells compared with cells transfected with a nontargeting siRNA (Fig. 1*F*). Furthermore, high-resolution confocal imaging of OMA1 siRNA-treated cells revealed mitochondrial heterogeneity in the MOMP response: in individual BAX/OMI double-positive cells, some mitochondria contained translocated BAX (*green puncta*) and lost OMI, whereas other mitochondria lacked BAX and retained OMI (*red puncta*) (Fig. 1*D*).

A BAX siRNA pool (which targets both endogenous BAX and ectopic Venus-BAX expression) potently inhibited etoposide-induced apoptosis and markedly reduced Venus-BAX fluorescence (Fig. 1, *C* and *F*). However, knockdown of BAK did not inhibit apoptosis, indicating that in these etoposide-treated cells, MOMP was predominantly BAX dependent. Transfection efficiency in high-throughput screening experiments was verified using a cell death–inducing transfection marker (siTOX) that consistently produced ∼90% loss of cells within 2 days post-transfection (not shown). Efficient knockdown of other proteins produced by siRNA pools was confirmed in supporting experiments (Fig. S1*A*).

### The siRNA screen uncovers potential regulators of MOMP

The image-based assay was used to screen 1318 gene-specific siRNA pools. We ranked gene scores in three groups corresponding to the cell phenotype categories noted previously: genes whose knockdown (I) increased the percentage of Venus-BAX puncta, (II) decreased the percentage of cells with Venus-BAX puncta, or (III) increased the percentage of BAX/OMI double-positive cells. When we applied a 10% cutoff in the score distribution range, we identified ∼200 initial “hits” (with scores approximately threefold to fivefold above or below controls for the categories I and II). Hits in category III were from the top 10% tail of the score distribution (Fig. 1*G*). For a secondary screen, we chose to reassay 95 of the primary screen hits, based on their effect scores and our judgment concerning their biological interest (we decided not to pursue some “housekeeping” genes). In this secondary screen, we tested the four siRNAs from each siRNA pool individually. To reduce the likelihood of off-target effects, we required at least three of the four individual siRNAs to give concordant results (34). Applying this more stringent criterion yielded a final list of 63 candidate genes (Table S2). Several hits in categories I and II were consistent with previous reports. For example, the effect of siRNA targeting Bcl-2/adenovirus E1B 19-kDa interacting protein 3 (BNIP3) (category II) is consistent with the known cell death–promoting activity of this protein (35). Acting in the opposite manner, the siRNAs scoring in category I increased the percentage of cells undergoing MOMP. For example, several candidate genes in this group are required for metabolism of the mitochondrial lipid, cardiolipin (phospholipid scramblase 3 [PLSCR3], PRELI domain–containing protein 1 [PRELIDI], and TP53-regulated inhibitor of apoptosis 1 [TRIAP1]). Although cardiolipin is important for BAX pore formation (6, 36), in certain paradigms, cardiolipin deficiency potentiates the release of apoptogenic proteins (37, 38). In particular, the deficiency of p53-regulated protein TRIAP1 impaired cardiolipin level in mitochondria, compromised bioenergetics, and potentiated cytochrome *c* release (38). Potential novel regulators of MOMP include metabolic enzymes, long-chain acyl-coenzyme A dehydrogenase, which catalyzes one of the early steps in the circle of mitochondrial beta-oxidation of fatty acids, and fumarylacetoacetate hydrolase domain–containing protein 1 with putative oxaloacetate decarboxylase activity in mitochondria. The roles of these proteins in mitochondrial metabolism and cell senescence are emerging (39, 40), and their inhibitory effects on MOMP could be of interest for further investigations. In this study, we did not further pursue the hits in categories I and II but focused on the unique phenotype produced by the hits in category III. Of note, in nearly all cells containing both BAX and OMI puncta, BAX and OMI were not colocalized, implying that some mitochondria within a cell had undergone MOMP, whereas others had not (similar to the phenotype shown in Fig. 1*D*). Perhaps the most striking outcome of our screen is that a majority of hits in category III are related to the MQC system.

### The interplay of MQC and MOMP

The concept of MQC is that mitochondrial dynamics (fission and fusion) work in tandem with mitophagy (the autophagic elimination of dysfunctional mitochondrial fragments) to maintain mitochondrial structural and functional integrity. If defective mitochondria cannot be repaired through fusion with functional organelles, they are prone to excessive fragmentation and elimination by mitophagy. It is postulated that asymmetrical fission segregates defective mitochondria, making the mitochondrial population inherently heterogeneous. These isolated small mitochondria are then redirected into a preautophagic pool (41, 42). In cells dysfunctional for mitochondrial dynamics or mitophagy, damaged mitochondria would be predicted to accumulate, and the organelles would become abnormally heterogeneous with respect to bioenergetic function and the distribution of proteins involved in MOMP (43, 44). Our screen tended to confirm this prediction.

### Downregulation of mitophagy-related genes promotes MOMP heterogeneity

Among the hits in category III, both ubiquitin-dependent (*e.g.*, autophagy-related 12 [ATG12] and mitochondrial E3 ubiquitin protein ligase 1 [MUL1]) and ubiquitin-independent (*e.g.*, FUN14-domain containing 1 [FUNDC1], BNIP3-like [BNIP3L], biogenesis of lysosome-related organelles complex 1 subunit 1 [BLOC1S1]/general control of amino acid synthesis 5-like 1 [GCN5LI], TRIAP1, and membrane-associated ring finger (C3HC4) 5 [MARCH5]) mitophagy mechanisms (45, 46, 47, 48) were represented (Table 1). ATG12 is ubiquitin-like protein that is integral for general autophagy and has additional roles in apoptosis and cell survival (49). For example, ATG12 deficiency compromises mitochondrial function and promotes cellular oncogenic transformation (50). One of the top-scoring hits was BLOC1S1/GCN5LI, which is reported to have multiple functions, including the coordinate regulation of mitochondrial biogenesis and mitophagy, through protein acetylation (51). BNIP3L/NIX (originally described as a BCL-2-related proapoptotic protein) and FUNDC1 directly interact with the light chain 3 protein in the autophagosome formation pathway and promote mitophagy facilitated by hypoxic conditions (48, 50, 52, 53).

**Table 1:** A list of genes whose downregulation increased mitochondrial heterogeneity (BAX/OMI-positive phenotype) in siRNA screen

Gene	Score	Function
RNF5	85.3	E3 ubiquitin ligase; ER quality control; translocates from ER to mitochondrial in antiviral response; controls UPR, **autophagy**, antitumor immunity (71, 72, 73, 74)
LETM1	78.8	Mitochondrial Ca^2+^ and/or K^+^ transport; implicated in assembly of ETC supercomplexes and maintenance of tubular **mitochondrial morphology** (64, 75, 76)
MARCH5	64	E3 ubiquitin ligase; regulates **mitophagy** and **mitochondrial dynamics***via* targeting FUNDC1, MFN1/2, and Fis1; involved in MAVS signaling (77, 78, 79)
FUNDC1	59.3	Regulation of LC3-dependent **autophagy** and **parkin-independent mitophagy**; a MARCH5 target; regulates **MQC** in hypoxia, **interacts with Drp1** (46, 77, 80)
DISC1	50.3	Disrupted-in-schizophrenia-1; enriched in MAM; controls mitofilin stability, regulates **mitochondrial trafficking**, Ca^2+^ dynamics, and **bioenergetics** (81, 82, 83)
ULK1	48.3	**Autophagy**-activating kinase; phosphorylates FUNDC1; interacts with Bcl-2-L13 and LC3B to induce **mitophagy**; negatively regulated by mTOR (84, 85, 86)
**ATG12**	42.8	Integral component of general and selective **autophagy**, binding partner of ATG5; promotes **apoptosis**; regulates **mitochondrial biogenesis** (14, 50, 87)
**RMDN3**	39.5	Encodes tyrosine phosphatase–interacting protein-51 (PTPIP51); controls mitochondria–ER tethering, ER stress, **autophagy**, **parkin-dependent mitophagy** (54, 55, 56)
NR2C2	38.7	Nuclear receptor/transcriptional regulator (also known as TR4); DNA repair function; KO promotes tumorigenesis, **complex I deficiency**, myopathy (88, 89, 90)
IER3	37	Early response gene, multifunctional; positive and negative **apoptosis** regulator, tumor-suppressive activity; interacts with Mcl-1; **modulates complex V** (91, 92, 93)
SLC25A14	33.3	Encodes UCP5; mitochondrial metabolite/anion transporter; KD **decreases ΔΨ**; implicated in ROS production, and DJ-1-dependent neuroprotection (94, 95, 96)
PHB	33.3	Prohibitin; controls **ETC protein stability**, **OMA1/OPA1-dependent fusion** and cristae morphogenesis, **apoptosis**, cancer cell proliferation (97, 98, 99)
MRPL12	33	Mitochondrial ribosomal protein; regulates mitochondrial gene expression; upregulated in cancer; mutations are linked to a mitochondrial disease (100, 101, 102)
**BNIP3L**	30	Atypical **proapoptotic** Bcl-2 family protein, multifunctional, mediates hypoxia-activated **autophagy and mitophagy** with effects on **mitochondrial fission** (48, 52, 103)
GPER1	24	G protein–coupled estrogen receptor
TMEM127	24	Endosome-associated tumor suppressor gene, linked to various cancers; regulates **Rab5-mediated endosomal fusion** (104); mitochondrial function is unknown
ATCAY	23.7	Encodes caytaxin, a brain-specific kinesin-interacting protein; involved in kinesin-dependent **mitochondrial motility** on microtubules (105)
TRIAP1	22	p53-regulated **antiapoptotic factor**, linked to tumorigenesis; KD promotes **MOMP and fission**; lipid transfer activity, cardiolipin biosynthesis (38, 106, 107)
C19orf12	16.3	An orphan mitochondrial protein whose loss of function is linked to neurodegenerative diseases; involved in lipid metabolism, stimulates **autophagy** (108, 109)
SPATA19	15	Spermatogenesis-associated 19; important for sperm motility by regulating mitochondrial structure and function (110)
BLOC1S1	9.5	General control of amino acid synthesis 5 like-1 (GCN5L1/BLOC1S1); endosome–lysosomal function; mitochondrial protein acetylation and **mitophagy** (51)
TRAC1	7	Essential for kinesin 1-dependent **mitochondrial trafficking** (57, 58)

Abbreviations: ETC, electron transport chain; MAM, mitochondria-associated ER membrane; MAVS, mitochondrial antiviral-signaling protein; mTOR, mammalian target of rapamycin; UPR, unfolded protein response.

MQC functions (mitochondrial dynamics, mitophagy) of the hits are highlighted in *bold*. Other highlighted functions that can affect mitochondrial heterogeneity include regulation of mitochondrial respiration and apoptosis. Genes in boldface (RMDN3, ATG12, and BNIP3L) were selected for further experiments. Numbers indicate assay scores obtained in the secondary screen. Other secondary screen hits are listed in Table S2.

### Other MQC-related hits in category III

Besides well-defined components of mitophagy pathways, our screen yielded multiple candidates that could affect MQC as part of their function. For example, protein tyrosine phosphatase–interacting protein 51 (encoded by the regulator of microtubule dynamics protein 3 [RMDN3] gene) was shown to be important for mitochondria–endoplasmic reticulum (ER) tethering, and therefore, its putative role in MQC and apoptosis could be linked to multiple functions regulated by ER–mitochondrial contacts, such as ER stress–induced Ca^2+^ release (54), autophagosome formation, and mitochondrial fission (55, 56).

Another functional group represented by multiple hits in the screen (ATCAY, disrupted-in-schizophrenia 1 [DISC1], trafficking protein kinesin–binding 1, and spermatogenesis-associated 19 [SPATA19]) involves mitochondrial motility on microtubules, in which motor proteins such as kinesin 1 interact with certain mitochondrial membrane proteins, for example, mitochondrial Rho GTPase 1 (57, 58). We suspect that these genes appeared as hits in our screen because microtubule-based motility is important for mitochondrial fusion, including the transient fusion event known as “kiss and run” (59, 60). Since fusion is a key element of MQC, a loss of mitochondrial motility would be expected to increase the functional heterogeneity of the mitochondrial population within each cell.

Other interesting candidates include multifunctional E3 ubiquitin ligases ring finger protein 5 (RNF5) and MARCH5. Mitochondrial targets and pathways regulated by ring finger protein 5 remain to be identified. MARCH5 reportedly regulates activities of mitofusin-1 (MFN1) and dynamin-related protein 1 (DRP1) or its outer membrane receptor mitochondrial fission factor, the key mediators of mitochondrial fusion and fission, respectively (61, 62, 63). However, DRP1 and MFN themselves did not show significant effects in our screen, perhaps because of the redundancy. Among multifunctional proteins that may influence mitochondrial dynamics indirectly, leucine zipper-EF-hand containing transmembrane protein 1 (LETM1) is the mitochondrial Ca^2+^/H^+^ antiporter with pleiotropic effects on ATP production, mitophagy, cristae structure, and mitochondria morphology (64). In particular, mitochondria lacking LETM1 are prone to undergo DRP1-independent fission (65). In our screen, LETM1 knockdown also increased mitochondrial fragmentation (not shown) as well as MOMP heterogeneity. Other candidate genes in category III are highlighted in Table 1. Overall, our screen suggests that a deficiency in MQC disturbs the normal apoptotic response and, in particular, promotes heterogeneous MOMP.

### An assay to measure clonogenic cell survival despite caspase activation

**Figure 2:**
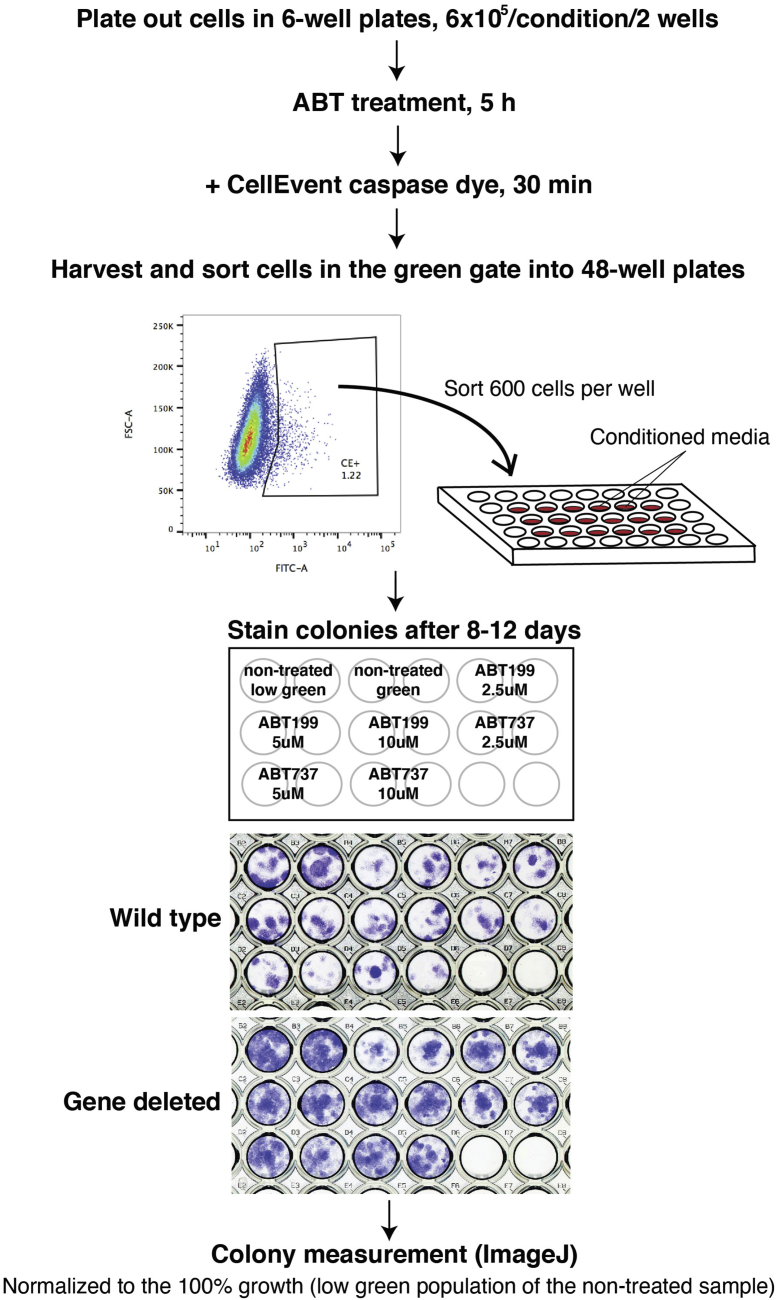
**Cell-based “minority MOMP” assay.** Cells were treated with ABT199 or ABT737 for 5 h, and a caspase dye was added for the final 30 min. Cells were harvested, and 600 cells from the FITC-positive gate were sorted into 48-well plates containing the conditioned media. Grown colonies were stained with crystal violet, and colony areas were measured using the macro developed by Guzman *et al*. (23) (see the Experimental procedures section). MOMP, mitochondrial outer membrane permeabilization.

Based on the number of hits our screen being involved in MQC, we hypothesized that MQC-dependent mitochondrial heterogeneity could result in an increased frequency of “minority MOMP.” In this scenario, when a minor fraction of mitochondria undergoes MOMP, the resulting sublethal caspase activation leads to DNA damage and promotes oncogenesis (17). To test our hypothesis, we developed a clonogenic survival assay based on that described by Ichim *et al*. (17) (Fig. 2). In this assay, we induced apoptosis with a BH3-mimetic compound, ABT-737 or ABT-199, incubated the cells with a fluorescent caspase-reporter compound, and used fluorescence-activated cell sorting to sort out cells that exhibited caspase activation. We then plated the cells and, after several days in culture, quantified the percentage of cells that survived and formed colonies.

We chose the BH mimetics for this assay, instead of etoposide used in the screen, for their well-defined molecular targets in MOMP (66, 67) and no known toxic effects on other apoptotic pathways. Ichim *et al.* ((17); Fig. 1*E*) demonstrated that these mimetics induced minority MOMP in HeLa and U2OS cells. Therefore, we reasoned that these reagents would cause heterogenous MOMP as in the screen.

**Figure 3:**
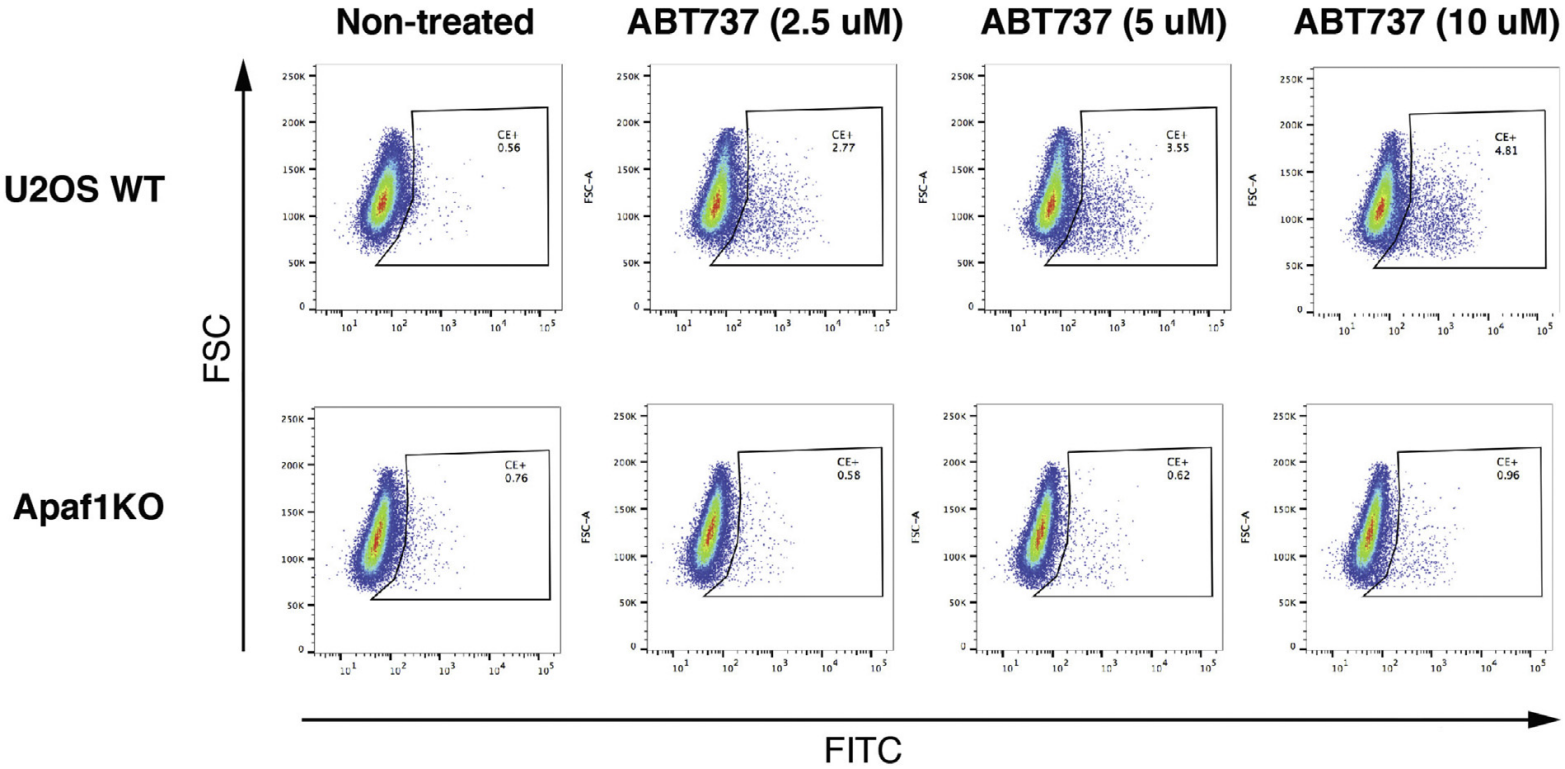
**The caspase reporter dye faithfully detects activated caspases**. Dot plots of WT U2OS and APAF-1 CRISPR KO cells stained with a caspase dye (CellEvent; Life Technologies) after treatment with ABT737 at 2.5, 5, or 10 μM for 5 h. APAF-1, apoptotic protease-activating factor-1.

In order to validate the faithfulness of the caspase reporter dye, we compared the staining in APAF-1 CRISPR KO U2OS cells (obtained from Dr Stephen Tait, University of Glasgow) to that of their parental WT control cells. APAF-1 is required to activate caspase-9 and subsequently the effector caspase-3 and caspase-7, following the MOMP-dependent release of cytochrome *c*, SMAC, and OMI. As shown in Figure 3, only a trace amount of cells were stained by the dye in the APAF-1 KO cell population treated with a BH3 mimetic, ABT-737. In contrast, the WT cells showed a population of cells stained with the dye, indicating that the caspase reporter was faithful in detecting caspase activation in the cells. Based on the study by Ichim *et al.* (17), it is reasonable to assume that survival of cells from this caspase-activated population indicates the occurrence of minority MOMP.

**Figure 4:**
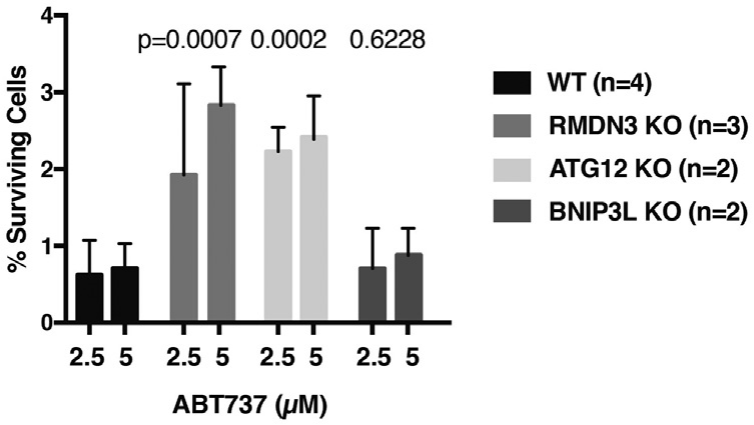
**Heterogeneous MOMP promotes cell survival after caspase activation.** RMDN3, ATG12, and BNIP3L, candidate genes from the screen for the BAX/OMI-positive phenotype, were targeted by CRISPR in U2OS cells, and corresponding KO cells were subjected to the clonogenic survival assay. The percentages of surviving cells were plotted. *p* Values above the bar graphs show the difference in survival between the WT U2OS cells and each KO line. ATG12, autophagy-related 12; BAX, BCL2-associated X; MOMP, mitochondrial outer membrane permeabilization; RMDN3, regulator of microtubule dynamics protein 3.

**Figure 5:**
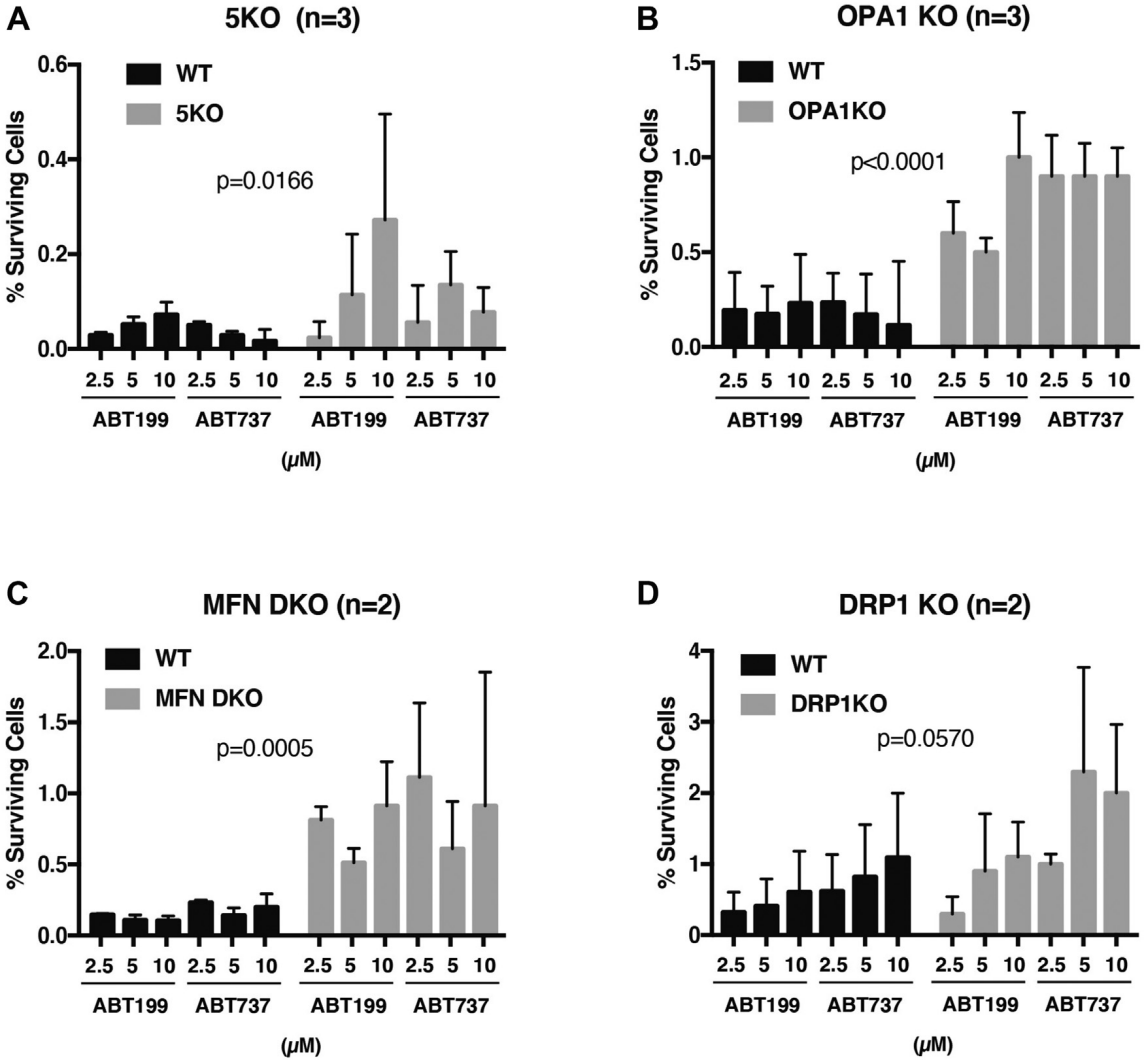
**Minority MOMP is enhanced by deletion of mitophagy-related or mitochondria dynamics–related genes.***A*, mitophagy defective penta-KO cells and their parental WT HeLa cells. *B*, OPA1 KO MEFs and their matched WT MEFs. *C*, MFN DKO MEFs and their matched WT MEFs. *D*, DRP1 KO MEFs and DRP1-positive WT MEFs used in the MFN set in (*C*). n denotes the number of independent experiments that were averaged. Error bars represent standard deviation. DKO, double KO; DRP1, dynamin-related protein 1; MEF, mouse embryonic fibroblast; MFN, mitofusin; MOMP, mitochondrial outer membrane permeabilization; OPA1, optic atrophy 1.

Given the challenging nature of the assay because of the sensitivity to temperature fluctuations, the cell density as well as the distribution in the wells (see also in Experimental procedures section), we only collected the data from the experiments flawlessly performed. Therefore, the data points for statistical analysis shown in Figures 4 and 5 were based only on two to three independent experiments. Further independent verification of these results will provide higher confidence in our findings.

### MQC deficiency promotes minority MOMP

To test further whether MQC is important to mitigate minority MOMP, we used the clonogenic assay described previously to investigate the effects of deleting selected candidate genes from our screen as well as other known MQC factors. We generated CRISPR cells (U2OS) with perturbed expression of ATG12, RMDN3 (protein tyrosine phosphatase–interacting protein 51), and BNIP3L. Knockdown of each of these genes produced MOMP heterogeneity in our imaging assay (Table 1). To obtain stable KO cells, we generated CRISPR-edited cell pools and derived clonal populations from single cells (as described in the Experimental procedures section). Validation of the CRISPR-based gene-editing efficiency is shown in Fig. S2. As shown in Figure 4, we found that cells depleted of either RMDN3 or ATG12 showed significantly higher post-MOMP survival than WT cells. However, BNIP3L KO did not show an increase in minority MOMP. Reasons for this are unknown but could be related to other apoptosis-related activities of BNIP3L.

To examine further the hypothesis that compromised MQC promotes minority MOMP, we performed experiments using previously derived cells in which genes known to be directly involved in mitochondrial dynamics or mitophagy had been deleted, namely MFN1 and MFN2 double KO, OPA1 KO, DRP1 KO mouse embryonic fibroblasts (MEFs), and “penta-KO” HeLa cells that had been shown to be deficient in mitophagy because of the deletion of five mitophagy receptor genes (21). As shown in Figure 5, all these MQC-deficient cells exhibited an enhancement of cell survival in our clonogenic assay, compared with their matched controls. The effect in DRP1 KO cells was not statistically significant; in this case, a weaker effect could reflect the slowing of cell proliferation that was reported with DRP1 deletion (68). Taken together, the loss of these genes, important for MQC, promoted the proliferative survival of caspase-engaged cells, again implying an increased frequency of minority MOMP.

## Conclusion

Taken together, our data allow us to conclude that the MQC system is critical for mitigating the phenomenon of minority MOMP. This result has implications for oncogenesis; even a small increase in cell viability resulting from sublethal caspase activation could potentially raise the frequency of oncogenic cell transformation (69, 70). Therefore, treatments developed to limit minority MOMP could improve the cytotoxic effects of anticancer treatments. For example, proteins that are known to inhibit mitophagy, such as seven in absentia homolog 3 (21), are potential therapeutic targets. Also, we predict, based on our observation that placing sorted caspase-engaged cells on ice eliminated their ability to survive clonogenicity (see the Experimental procedures section), that cryotherapy could prevent minority MOMP when used in combination with proapoptotic cancer therapeutics such as BH3-mimetic drugs. In conclusion, our study provides new insights into the mechanisms of aberrant apoptosis that are important for developing therapeutic strategies.

## Experimental procedures

### Cell lines

HeLa cells stably expressing Venus-BAX and OMI-mCherry cells (20) were constructed by the laboratory of Douglas Green (Department of Immunology, St Jude Children Hospital). U2OS cells with APAF-1 CRISPR KO were obtained from Dr Stephen Tait (Beatson Institute) (17) and MFN 1 and MFN2 double-KO cells were obtained from Dr David Chan (California Institute of Technology). Immortalized OPA1 KO and WT MEFs were obtained from American Type Culture Collection (deposited by Dr David Chan). DRP1 KO MEFs were provided by Dr Stefan Strack (University of Iowa); HeLa cells lacking five mitophagy receptors (TAX1BP1, NDP52, OPTN, NBR1, p62; Penta KO) were provided by Dr Richard Youle (the National Institutes of Health) (21). Unless indicated otherwise, cells were maintained in Dulbecco's modified Eagle’s medium (DMEM; Life Technologies) containing 10% fetal bovine serum (FBS) (GeminiBio) and 100 units/ml penicillin/streptomycin at 37 ^°^C with 5% CO_2_.

### siRNA screen: cell transfections and treatments

For the primary screen, gene-specific siRNA pools targeting 1318 mitochondria-annotated genes (Table S1) were cherry-picked from a Dharmacon genome-wide siRNA library (siGENOME). The list of genes was generated based on a “mitochondria/mitochondrial membranes” queries in the National Institutes of Health compiled databases (http://www.ncbi.nlm.nih.gov/gene). Pools of four individual gene-specific siRNAs and two nontargeting siRNAs were arrayed in 384-well master plates. Each plate also contained Dharmacon siGENOME TOX and siGlo Red oligonucleotides as transfection indicators. In addition, an siRNA pool targeting BAX was used as a positive control for the inhibition of apoptosis and BAX puncta formation. For reverse transfection, 4.4 μl siRNA picked from each 1 μM stock siRNA solution in the master plate was mixed with 30.6 μl prediluted Lipofectamine RNAiMAX transfection reagent (Life Technology). RNAiMAX reagent was diluted 47 times in Gibco Opti-MEM reduced serum medium. The transfection mixtures (35 μl/well) were incubated for 20 min at room temperature in a 384-well mixing plate. During the incubation period, Venus-BAX/OMI-mCherry HeLa cells were harvested by trypsinization and resuspended in antibiotic-free DMEM with 10% FBS at 30,000 cells per ml. After incubation, lipid–siRNA complexes were dispensed into triplicate tissue/culture Costar black-wall clear bottom 384-well plates (10 μl/well); siRNAs targeting the same gene were separated in the different plates. Plate handling, transfection reagent/siRNA mixing, and dispensing were performed with Hamilton Star Automated Liquid Handler contained within a Baker BioPROTECT class II biosafety cabinet. Cells were added at 40 μl (1200 cells) per well on top of the transfection mixture; final concentration of siRNA was 25 nM in the 50 μl per well volume. The plates were centrifuged at low speed (∼300*g*) for 1 min and transferred to a humidified CO_2_ incubator (37 °C). To minimize plate edge effects, the wells in two rows and columns at the plate edges were not used for transfections and contained only medium with cells. After ∼48 h, the medium was aspirated and replaced with fresh culture medium containing 400 μM etoposide (Sigma–Aldrich) and 20 μM of the caspase inhibitor Q-VD (Q-VD-OPH; SM Biochemicals LLC). Control wells were left untreated (no etoposide). By the time of etoposide treatment, most cells transfected with TOX siRNA had already shown morphological changes consistent with cell death, indicating efficient transfection. Following 24 h of etoposide treatment, the medium was removed, and cells were fixed with 0.5% glutaraldehyde as described previously (22). After two washes with PBS, cells were stained with Hoechst-33342 (Molecular Probes) diluted 1000 times in PBS. Plates were then washed twice with PBS, filled with 50 μl PBS per well, sealed, and stored at 4 °C. For the secondary screen, four individual siGENOME siRNAs per gene were obtained separately from Dharmacon to target 95 gene candidates chosen from the primary screen. Individual siRNAs were arrayed in replicate 384-well plates, and the experiments were conducted as described previously.

### High-throughput image acquisition and analysis

Image collection and processing were done using the Molecular Devices MetaXpress High Content Image Acquisition platform. Images of the cells were acquired in an Image Xpress Micro device at 20× magnification with the following filters: 4′,6-diamidino-2-phenylindole (5060B), for Hoechst nuclear staining; YFP (2427A), for Venus-BAX fluorescence; and Texas Red (4040B), for OMI-mCherry fluorescence (numbers indicate the Semrock part number for the filters). Images were collected from 16 sites clustered in the center of the well. In our assay development, the punctate patterns of Venus-BAX translocation and OMI-mCherry retention in mitochondria were analyzed using the granularity module in MetaXpress (version 5.1) software (Molecular Devices). Individual cells were identified based on nuclear segmentation (4′,6-diamidino-2-phenylindole channel images). Z′ factor for the BAX-positive or BAX-negative phenotype assay was calculated as 0.85 based on etoposide-treated and untreated conditions as positive and negative controls, respectively.

High-throughput analysis of primary and secondary screen data was done with a customized algorithm (created as a “journal macro” in MetaXpress) developed for automated phenotype quantification. We configured image analysis to count the percentage of cells that satisfied either of two different criteria: (1) a given cell contains Venus-BAX granules of diameters within the expected diameter range, with total intensity above a certain threshold or (2) a given cell contains both suprathreshold Venus-BAX and OMI-mCherry granules (not necessarily colocalized) within the appropriate diameter range. The thresholds were adjusted for stringency to limit false-positive scores from background fluorescence and debris. Plate-to-plate variability was minimal for BAX foci and acceptably low for cells double positive for BAX and OMI granules.

### Confocal microscopy

Confocal images of Venus-BAX/OMI-mCherry cells were acquired with a 60× oil immersion objective on an Olympus FluoView FV10i automated confocal laser scanning microscope (Olympus Scientific Solutions America Corp).

### CRISPR/Cas9-mediated gene depletion

CRISPR experiments were performed using modified synthetic single-guide RNAs (sgRNAs) from Synthego. Target sequences for guide RNAs were selected with the Synthego CRISPR design tool. RNA oligonucleotides were reconstituted in 10 mM Tris–HCl, 1 mM EDTA, pH 8.0 buffer according to recommendations from the manufacturer. Ribonucleoprotein complexes were formed from sgRNA and recombinant Cas9 two nuclear localization signal proteins (New England Biolabs) mixed at an sgRNA to Cas9 ratio of 4.5:1. After 20 to 30 min of incubation at room temperature, assembled ribonucleoproteins were delivered into cells by electroporation using a ThermoNeon device and 10 μM tips (Thermo Fisher Scientific). For one sample (∼2 × 10^5^ cells), 3 μl of sgRNA (from 30 mM stock solution) were mixed with 1.5 μl Cas9 protein (from 20 μM stock solution). U2OS cells were electroporated at 1230 v/10 ms/4 (pulse voltage, width, and pulse number) settings. Immediately after electroporation, cells were transferred to 12-well plate with prewarmed antibiotic-free DMEM with 10% FBS. After 2 to 3 days of incubation, a portion of the control (unedited) and CRISPR cells were harvested for genomic DNA isolation using a QIAGEN genomic DNA purification kit. The remaining cells were left in culture for further propagation and clonal selection. Genomic DNA concentrations were measured using a NanoDrop instrument (Thermo Fisher Scientific). Primer design and PCR amplification of the edited region were done according to Synthego recommendations. Sanger sequencing of PCR amplicons was performed at Genewiz or Eton Bioscience; the resulting DNA sequencing chromatograms were analyzed using the Inference of CRISPR Edits algorithm (Synthego). Inference of CRISPR Edits analysis of CRISPR-edited genomic regions typically demonstrated at least 70% editing efficiency (*i.e.*, 70% KO cells in the pool); sgRNA sequences used are shown in Fig. S2. For clone selection, CRISPR cells were expanded for 2 to 3 additional days, harvested, and subjected to sorting into 96-well plates using BD FACSAria-3 or FACSAria-4 Fusion instruments; typically, one to four single cells were dispensed into one well. After clonal expansion, cells were analyzed for KO efficiency as described previously. For each gene of interest, two clones with verified KO were combined for use in further experiments.

### Assay for clonogenic survival after caspase activation *via* MOMP (outlined in Fig. 2)

Cells (CRISPR KO U2OS cells and MEFs [KO and matched WT]) were plated out at 3 × 10^5^ per well containing 1 ml media in 6-well plates the day before the experiment, two wells per condition. The cells were then treated with ABT-199 or ABT-737 at 2.5, 5, or 10 μM for 5 h. In the last 30 min of incubation, a caspase reporter dye, CellEvent Caspase3/7 Green (Life Technology; R37111), was added at 30 μl per well. Cells were harvested with trypsin/EDTA and washed with medium and PBS. Finally, cells were resuspended in 300 μl of sorting buffer consisting of 1% bovine serum albumin in PBS containing 20 μM of Q-VD, where Q-VD was included to stop the caspase reaction. We noticed that, when these caspase-activated cells were left at room temperature or 4 ^°^C for more than 30 to 40 min, their ability to survive was compromised. Therefore, care was taken to sort the cells immediately after harvesting. To minimize artifacts from sample handling delay, we operated two cell sorters (FACSAria; BD) simultaneously for WT and gene-defective cells. Also, the sorting chamber and the plate holder were kept at 37 ^°^C during the run to prevent temperature shock to the cells. Six hundred cells within the population gated for green fluorescence were sorted in duplicate into a 48-well plate containing 300 μl of conditioned medium per well. The cells were grown for 8 to 12 days, during which an additional 500 μl of conditioned medium was added at days 4 to 5. Colonies were then stained with 6% glutaraldehyde containing 0.5% crystal violet. Nontreated cells distributed in the no-green gate were sorted as aforementioned and used as a control representing 100% growth. Because individual cell colonies tended to merge over time, we measured the total area occupied by colonies, using the ImageJ macros developed by Guzman *et al.* (23). The percentage of surviving caspase-engaged cells in the total population was normalized against the 100% growth control. The data are summarized from two to three independent experiments, as indicated. *p* Values were calculated using the mean and the standard deviation in each set of KO and WT cells with two-way ANOVA analysis using Prism 7 (GraphPad Software, Inc).

## Data availability

All representative data are contained within the article.

## Supporting information

This article contains supporting information.

## Conflict of interest

The authors declare that they have no conflicts of interest with the contents of this article.

## References

[1] Chipuk J.E., Moldoveanu T., Llambi F., Parsons M.J., Green D.R. (2010). The BCL-2 family reunion. Mol. Cell.

[2] Youle R.J., Strasser A. (2008). The BCL-2 protein family: Opposing activities that mediate cell death. Nat. Rev. Mol. Cell Biol..

[3] Gillies L.A., Du H., Peters B., Knudson C.M., Newmeyer D.D., Kuwana T. (2015). Visual and functional demonstration of growing Bax-induced pores in mitochondrial outer membranes. Mol. Biol. Cell.

[4] Kushnareva Y., Andreyev A.Y., Kuwana T., Newmeyer D.D. (2012). Bax activation initiates the assembly of a multimeric catalyst that facilitates bax pore formation in mitochondrial outer membranes. PLoS Biol..

[5] Kuwana T., Olson N.H., Kiosses W.B., Peters B., Newmeyer D.D. (2016). Pro-apoptotic Bax molecules densely populate the edges of membrane pores. Sci. Rep..

[6] Kuwana T., Mackey M.R., Perkins G., Ellisman M.H., Latterich M., Schneiter R., Green D.R., Newmeyer D.D. (2002). Bid, Bax, and lipids cooperate to form supramolecular openings in the outer mitochondrial membrane. Cell.

[7] Ader N.R., Hoffmann P.C., Ganeva I., Borgeaud A.C., Wang C., Youle R.J., Kukulski W. (2019). Molecular and topological reorganizations in mitochondrial architecture interplay during Bax-mediated steps of apoptosis. Elife.

[8] Nicholson D.W., Thornberry N.A. (1997). Caspases: Killer proteases. Trends Biochem. Sci..

[9] Bratton S.B., Salvesen G.S. (2010). Regulation of the Apaf-1-caspase-9 apoptosome. J. Cell Sci..

[10] Ricci J.E., Muñoz-Pinedo C., Fitzgerald P., Bailly-Maitre B., Perkins G.A., Yadava N., Scheffler I.E., Ellisman M.H., Green D.R. (2004). Disruption of mitochondrial function during apoptosis is mediated by caspase cleavage of the p75 subunit of complex I of the electron transport chain. Cell.

[11] Kushnareva Y., Newmeyer D.D. (2010). Bioenergetics and cell death. Ann. New York Acad. Sci..

[12] Lartigue L., Kushnareva Y., Seong Y., Lin H., Faustin B., Newmeyer D.D. (2009). Caspase-independent mitochondrial cell death results from loss of respiration, not cytotoxic protein release. Mol. Biol. Cell.

[13] Colell A., Ricci J.E., Tait S., Milasta S., Maurer U., Bouchier-Hayes L., Fitzgerald P., Guio-Carrion A., Waterhouse N.J., Li C.W., Mari B., Barbry P., Newmeyer D.D., Beere H.M., Green D.R. (2007). GAPDH and autophagy preserve survival after apoptotic cytochrome c release in the absence of caspase activation. Cell.

[14] Tait S.W., Green D.R. (2013). Mitochondrial regulation of cell death. Cold Spring Harb. Perspect. Biol..

[15] Gong Y.N., Crawford J.C., Heckmann B.L., Green D.R. (2019). To the edge of cell death and back. FEBS J..

[16] Tait S.W., Parsons M.J., Llambi F., Bouchier-Hayes L., Connell S., Muñoz-Pinedo C., Green D.R. (2010). Resistance to caspase-independent cell death requires persistence of intact mitochondria. Dev. Cell.

[17] Ichim G., Lopez J., Ahmed S.U., Muthalagu N., Giampazolias E., Delgado M.E., Haller M., Riley J.S., Mason S.M., Athineos D., Parsons M.J., van de Kooij B., Bouchier-Hayes L., Chalmers A.J., Rooswinkel R.W. (2015). Limited mitochondrial permeabilization causes DNA damage and genomic instability in the absence of cell death. Mol. Cell.

[18] Ni H.M., Williams J.A., Ding W.X. (2015). Mitochondrial dynamics and mitochondrial quality control. Redox Biol..

[19] Pickles S., Vigie P., Youle R.J. (2018). Mitophagy and quality control mechanisms in mitochondrial maintenance. Curr. Biol..

[20] Llambi F., Moldoveanu T., Tait S.W., Bouchier-Hayes L., Temirov J., McCormick L.L., Dillon C.P., Green D.R. (2011). A unified model of mammalian BCL-2 protein family interactions at the mitochondria. Mol. Cell.

[21] Lazarou M., Sliter D.A., Kane L.A., Sarraf S.A., Wang C., Burman J.L., Sideris D.P., Fogel A.I., Youle R.J. (2015). The ubiquitin kinase PINK1 recruits autophagy receptors to induce mitophagy. Nature.

[22] Kushnareva Y., Seong Y., Andreyev A.Y., Kuwana T., Kiosses W.B., Votruba M., Newmeyer D.D. (2016). Mitochondrial dysfunction in an Opa1(Q285STOP) mouse model of dominant optic atrophy results from Opa1 haploinsufficiency. Cell Death Dis..

[23] Guzman C., Bagga M., Kaur A., Westermarck J., Abankwa D. (2014). ColonyArea: An ImageJ plugin to automatically quantify colony formation in clonogenic assays. PLoS One.

[24] Goldstein J.C., Waterhouse N.J., Juin P., Evan G.I., Green D.R. (2000). The coordinate release of cytochrome c during apoptosis is rapid, complete and kinetically invariant. Nat. Cell Biol..

[25] Yamaguchi R., Lartigue L., Perkins G., Scott R.T., Dixit A., Kushnareva Y., Kuwana T., Ellisman M.H., Newmeyer D.D. (2008). Opa1-mediated cristae opening is Bax/Bak and BH3 dependent, required for apoptosis, and independent of Bak oligomerization. Mol. Cell.

[26] Wang T.S., Coppens I., Saorin A., Brady N.R., Hamacher-Brady A. (2020). Endolysosomal targeting of mitochondria is integral to BAX-mediated mitochondrial permeabilization during apoptosis signaling. Dev. Cell.

[27] Munoz-Pinedo C., Guío-Carrión A., Goldstein J.C., Fitzgerald P., Newmeyer D.D., Green D.R. (2006). Different mitochondrial intermembrane space proteins are released during apoptosis in a manner that is coordinately initiated but can vary in duration. Proc. Natl. Acad. Sci. U. S. A..

[28] Ehses S., Raschke I., Mancuso G., Bernacchia A., Geimer S., Tondera D., Martinou J.C., Westermann B., Rugarli E.I., Langer T. (2009). Regulation of OPA1 processing and mitochondrial fusion by m-AAA protease isoenzymes and OMA1. J. Cell Biol..

[29] Head B., Griparic L., Amiri M., Gandre-Babbe S., van der Bliek A.M. (2009). Inducible proteolytic inactivation of OPA1 mediated by the OMA1 protease in mammalian cells. J. Cell Biol..

[30] Jiang X., Jiang H., Shen Z., Wang X. (2014). Activation of mitochondrial protease OMA1 by Bax and Bak promotes cytochrome c release during apoptosis. Proc. Natl. Acad. Sci. U. S. A..

[31] Frezza C., Cipolat S., Martins de Brito O., Micaroni M., Beznoussenko G.V., Rudka T., Bartoli D., Polishuck R.S., Danial N.N., De Strooper B., Scorrano L. (2006). OPA1 controls apoptotic cristae remodeling independently from mitochondrial fusion. Cell.

[32] Scorrano L., Ashiya M., Buttle K., Weiler S., Oakes S.A., Mannella C.A., Korsmeyer S.J. (2002). A distinct pathway remodels mitochondrial cristae and mobilizes cytochrome c during apoptosis. Dev. Cell.

[33] Kushnareva Y.E., Gerencser A.A., Bossy B., Ju W.K., White A.D., Waggoner J., Ellisman M.H., Perkins G., Bossy-Wetzel E. (2013). Loss of OPA1 disturbs cellular calcium homeostasis and sensitizes for excitotoxicity. Cell Death Differ..

[34] Sigoillot F.D., King R.W. (2011). Vigilance and validation: Keys to success in RNAi screening. ACS Chem. Biol..

[35] Chinnadurai G., Vijayalingam S., Gibson S.B. (2008). BNIP3 subfamily BH3-only proteins: Mitochondrial stress sensors in normal and pathological functions. Oncogene.

[36] Landeta O., Landajuela A., Gil D., Taneva S., DiPrimo C., Sot B., Valle M., Frolov V.A., Basañez G. (2011). Reconstitution of proapoptotic BAK function in liposomes reveals a dual role for mitochondrial lipids in the BAK-driven membrane permeabilization process. J. Biol. Chem..

[37] Choi S.Y., Gonzalvez F., Jenkins G.M., Slomianny C., Chretien D., Arnoult D., Petit P.X., Frohman M.A. (2007). Cardiolipin deficiency releases cytochrome c from the inner mitochondrial membrane and accelerates stimuli-elicited apoptosis. Cell Death Differ..

[38] Potting C., Tatsuta T., König T., Haag M., Wai T., Aaltonen M.J., Langer T. (2013). TRIAP1/PRELI complexes prevent apoptosis by mediating intramitochondrial transport of phosphatidic acid. Cell Metab..

[39] Etemad S., Petit M., Weiss A.K.H., Schrattenholz A., Baraldo G., Jansen-Dürr P. (2019). Oxaloacetate decarboxylase FAHD1 - a new regulator of mitochondrial function and senescence. Mech. Ageing Dev..

[40] Softic S., Meyer J.G., Wang G.X., Gupta M.K., Batista T.M., Lauritzen H.P.M.M., Fujisaka S., Serra D., Herrero L., Willoughby J., Fitzgerald K., Ilkayeva O., Newgard C.B., Gibson B.W., Schilling B. (2019). Dietary sugars alter hepatic fatty acid oxidation *via* transcriptional and post-translational modifications of mitochondrial proteins. Cell Metab..

[41] Twig G., Elorza A., Molina A.J., Mohamed H., Wikstrom J.D., Walzer G., Stiles L., Haigh S.E., Katz S., Las G., Alroy J., Wu M., Py B.F., Yuan J., Deeney J.T. (2008). Fission and selective fusion govern mitochondrial segregation and elimination by autophagy. EMBO J..

[42] Shirihai O.S., Song M., Dorn G.W. (2015). How mitochondrial dynamism orchestrates mitophagy. Circ. Res..

[43] Chen H., Chomyn A., Chan D.C. (2005). Disruption of fusion results in mitochondrial heterogeneity and dysfunction. J. Biol. Chem..

[44] Weaver D., Eisner V., Liu X., Várnai P., Hunyady L., Gross A., Hajnóczky G. (2014). Distribution and apoptotic function of outer membrane proteins depend on mitochondrial fusion. Mol. Cell.

[45] Narendra D., Tanaka A., Suen D.F., Youle R.J. (2008). Parkin is recruited selectively to impaired mitochondria and promotes their autophagy. J. Cell Biol..

[46] Liu L., Feng D., Chen G., Chen M., Zheng Q., Song P., Ma Q., Zhu C., Wang R., Qi W., Huang L., Xue P., Li B., Wang X., Jin H. (2012). Mitochondrial outer-membrane protein FUNDC1 mediates hypoxia-induced mitophagy in mammalian cells. Nat. Cell Biol..

[47] Levine B., Yuan J. (2005). Autophagy in cell death: An innocent convict?. J. Clin. Invest..

[48] Hamacher-Brady A., Brady N.R. (2016). Mitophagy programs: Mechanisms and physiological implications of mitochondrial targeting by autophagy. Cell Mol. Life Sci..

[49] Subramani S., Malhotra V. (2013). Non-autophagic roles of autophagy-related proteins. EMBO Rep..

[50] Liu H., He Z., Germič N., Ademi H., Frangež Ž., Felser A., Peng S., Riether C., Djonov V., Nuoffer J.M., Bovet C., Mlinarič-Raščan I., Zlobec I., Fiedler M., Perren A. (2019). ATG12 deficiency leads to tumor cell oncosis owing to diminished mitochondrial biogenesis and reduced cellular bioenergetics. Cell Death Differ..

[51] Scott I., Wang L., Wu K., Thapa D., Sack M.N. (2018). GCN5L1/BLOS1 links acetylation, organelle remodeling, and metabolism. Trends Cell Biol..

[52] Youle R.J., Narendra D.P. (2011). Mechanisms of mitophagy. Nat. Rev. Mol. Cell Biol..

[53] Hanna R.A., Quinsay M.N., Orogo A.M., Giang K., Rikka S., Gustafsson Å.B. (2012). Microtubule-associated protein 1 light chain 3 (LC3) interacts with Bnip3 protein to selectively remove endoplasmic reticulum and mitochondria *via* autophagy. J. Biol. Chem..

[54] Stoica R., De Vos K.J., Paillusson S., Mueller S., Sancho R.M., Lau K.F., Vizcay-Barrena G., Lin W.L., Xu Y.F., Lewis J., Dickson D.W., Petrucelli L., Mitchell J.C., Shaw C.E., Miller C.C. (2014). ER-mitochondria associations are regulated by the VAPB-PTPIP51 interaction and are disrupted by ALS/FTD-associated TDP-43. Nat. Commun..

[55] Gomez-Suaga P., Paillusson S., Miller C.C.J. (2017). ER-mitochondria signaling regulates autophagy. Autophagy.

[56] Puri R., Cheng X.T., Lin M.Y., Huang N., Sheng Z.H. (2019). Mul1 restrains Parkin-mediated mitophagy in mature neurons by maintaining ER-mitochondrial contacts. Nat. Commun..

[57] Henrichs V., Grycova L., Barinka C., Nahacka Z., Neuzil J., Diez S., Rohlena J., Braun M., Lansky Z. (2020). Mitochondria-adaptor TRAK1 promotes kinesin-1 driven transport in crowded environments. Nat. Commun..

[58] Zhao Y., Song E., Wang W., Hsieh C.H., Wang X., Feng W., Wang X., Shen K. (2021). Metaxins are core components of mitochondrial transport adaptor complexes. Nat. Commun..

[59] Lee C.A., Chin L.S., Li L. (2018). Hypertonia-linked protein Trak1 functions with mitofusins to promote mitochondrial tethering and fusion. Protein Cell.

[60] Liu X., Weaver D., Shirihai O., Hajnóczky G. (2009). Mitochondrial 'kiss-and-run': Interplay between mitochondrial motility and fusion-fission dynamics. EMBO J..

[61] Park Y.Y., Lee S., Karbowski M., Neutzner A., Youle R.J., Cho H. (2010). Loss of MARCH5 mitochondrial E3 ubiquitin ligase induces cellular senescence through dynamin-related protein 1 and mitofusin 1. J. Cell Sci..

[62] Nakamura N., Kimura Y., Tokuda M., Honda S., Hirose S. (2006). MARCH-V is a novel mitofusin 2- and Drp1-binding protein able to change mitochondrial morphology. EMBO Rep..

[63] Cherok E., Xu S., Li S., Das S., Meltzer W.A., Zalzman M., Wang C., Karbowski M. (2017). Novel regulatory roles of Mff and Drp1 in E3 ubiquitin ligase MARCH5-dependent degradation of MiD49 and Mcl1 and control of mitochondrial dynamics. Mol. Biol. Cell.

[64] Li Y., Tran Q., Shrestha R., Piao L., Park S., Park J., Park J. (2019). LETM1 is required for mitochondrial homeostasis and cellular viability (Review). Mol. Med. Rep..

[65] Dimmer K.S., Navoni F., Casarin A., Trevisson E., Endele S., Winterpacht A., Salviati L., Scorrano L. (2008). LETM1, deleted in Wolf-Hirschhorn syndrome is required for normal mitochondrial morphology and cellular viability. Hum. Mol. Genet..

[66] Oltersdorf T., Elmore S.W., Shoemaker A.R., Armstrong R.C., Augeri D.J., Belli B.A., Bruncko M., Deckwerth T.L., Dinges J., Hajduk P.J., Joseph M.K., Kitada S., Korsmeyer S.J., Kunzer A.R., Letai A. (2005). An inhibitor of Bcl-2 family proteins induces regression of solid tumours. Nature.

[67] van Delft M.F., Wei A.H., Mason K.D., Vandenberg C.J., Chen L., Czabotar P.E., Willis S.N., Scott C.L., Day C.L., Cory S., Adams J.M., Roberts A.W., Huang D.C. (2006). The BH3 mimetic ABT-737 targets selective Bcl-2 proteins and efficiently induces apoptosis via Bak/Bax if Mcl-1 is neutralized. Cancer Cell.

[68] Wakabayashi J., Zhang Z., Wakabayashi N., Tamura Y., Fukaya M., Kensler T.W., Iijima M., Sesaki H. (2009). The dynamin-related GTPase Drp1 is required for embryonic and brain development in mice. J. Cell Biol..

[69] Ichim G., Tait S.W. (2016). A fate worse than death: Apoptosis as an oncogenic process. Nat. Rev. Cancer.

[70] Liu X., He Y., Li F., Huang Q., Kato T.A., Hall R.P., Li C.Y. (2015). Caspase-3 promotes genetic instability and carcinogenesis. Mol. Cell.

[71] Kuang E., Qi J., Ronai Z. (2013). Emerging roles of E3 ubiquitin ligases in autophagy. Trends Biochem. Sci..

[72] Jeon Y.J., Khelifa S., Ratnikov B., Scott D.A., Feng Y., Parisi F., Ruller C., Lau E., Kim H., Brill L.M., Jiang T., Rimm D.L., Cardiff R.D., Mills G.B., Smith J.W. (2015). Regulation of glutamine carrier proteins by RNF5 determines breast cancer response to ER stress-inducing chemotherapies. Cancer Cell.

[73] Zhong B., Zhang L., Lei C., Li Y., Mao A.P., Yang Y., Wang Y.Y., Zhang X.L., Shu H.B. (2009). The ubiquitin ligase RNF5 regulates antiviral responses by mediating degradation of the adaptor protein MITA. Immunity.

[74] Li Y., Tinoco R., Elmén L., Segota I., Xian Y., Fujita Y., Sahu A., Zarecki R., Marie K., Feng Y., Khateb A., Frederick D.T., Ashkenazi S.K., Kim H., Perez E.G. (2019). Gut microbiota dependent anti-tumor immunity restricts melanoma growth in Rnf5(-/-) mice. Nat. Commun..

[75] Jiang D., Zhao L., Clapham D.E. (2009). Genome-wide RNAi screen identifies Letm1 as a mitochondrial Ca2+/H+ antiporter. Science.

[76] Tamai S., Iida H., Yokota S., Sayano T., Kiguchiya S., Ishihara N., Hayashi J., Mihara K., Oka T. (2008). Characterization of the mitochondrial protein LETM1, which maintains the mitochondrial tubular shapes and interacts with the AAA-ATPase BCS1L. J. Cell Sci..

[77] Chen Z., Liu L., Cheng Q., Li Y., Wu H., Zhang W., Wang Y., Sehgal S.A., Siraj S., Wang X., Wang J., Zhu Y., Chen Q. (2017). Mitochondrial E3 ligase MARCH5 regulates FUNDC1 to fine-tune hypoxic mitophagy. EMBO Rep..

[78] Yoo Y.S., Park Y.Y., Kim J.H., Cho H., Kim S.H., Lee H.S., Kim T.H., Sun Kim Y., Lee Y., Kim C.J., Jung J.U., Lee J.S., Cho H. (2015). The mitochondrial ubiquitin ligase MARCH5 resolves MAVS aggregates during antiviral signalling. Nat. Commun..

[79] Tanaka A., Cleland M.M., Xu S., Narendra D.P., Suen D.F., Karbowski M., Youle R.J. (2010). Proteasome and p97 mediate mitophagy and degradation of mitofusins induced by Parkin. J. Cell Biol..

[80] Wu W., Lin C., Wu K., Jiang L., Wang X., Li W., Zhuang H., Zhang X., Chen H., Li S., Yang Y., Lu Y., Wang J., Zhu R., Zhang L. (2016). FUNDC1 regulates mitochondrial dynamics at the ER-mitochondrial contact site under hypoxic conditions. EMBO J..

[81] Park Y.U., Jeong J., Lee H., Mun J.Y., Kim J.H., Lee J.S., Nguyen M.D., Han S.S., Suh P.G., Park S.K. (2010). Disrupted-in-schizophrenia 1 (DISC1) plays essential roles in mitochondria in collaboration with Mitofilin. Proc. Natl. Acad. Sci. U. S. A..

[82] Park S.J., Lee S.B., Suh Y., Kim S.J., Lee N., Hong J.H., Park C., Woo Y., Ishizuka K., Kim J.H., Berggren P.O., Sawa A., Park S.K. (2017). DISC1 modulates neuronal stress responses by gate-keeping ER-mitochondria Ca(2+) transfer through the MAM. Cell Rep..

[83] Norkett R., Lesept F., Kittler J.T. (2020). DISC1 regulates mitochondrial trafficking in a miro1-GTP-dependent manner. Front. Cell Dev. Biol..

[84] Murakawa T., Okamoto K., Omiya S., Taneike M., Yamaguchi O., Otsu K.A. (2019). A mammalian mitophagy receptor, Bcl2-L-13, recruits the ULK1 complex to induce mitophagy. Cell Rep..

[85] Kundu M. (2011). ULK1, mammalian target of rapamycin, and mitochondria: Linking nutrient availability and autophagy. Antioxid. Redox Signal..

[86] Wu W., Tian W., Hu Z., Chen G., Huang L., Li W., Zhang X., Xue P., Zhou C., Liu L., Zhu Y., Zhang X., Li L., Zhang L., Sui S. (2014). ULK1 translocates to mitochondria and phosphorylates FUNDC1 to regulate mitophagy. EMBO Rep..

[87] Rubinstein A.D., Eisenstein M., Ber Y., Bialik S., Kimchi A. (2011). The autophagy protein Atg12 associates with antiapoptotic Bcl-2 family members to promote mitochondrial apoptosis. Mol. Cell.

[88] Liu S., Lee Y.F., Chou S., Uno H., Li G., Brookes P., Massett M.P., Wu Q., Chen L.M., Chang C. (2011). Mice lacking TR4 nuclear receptor develop mitochondrial myopathy with deficiency in complex I. Mol. Endocrinol..

[89] Shen J., Lin H., Li G., Jin R.A., Shi L., Chen M., Chang C., Cai X. (2016). TR4 nuclear receptor enhances the cisplatin chemo-sensitivity via altering the ATF3 expression to better suppress HCC cell growth. Oncotarget.

[90] Lin S.J., Zhang Y., Liu N.C., Yang D.R., Li G., Chang C. (2014). Minireview: Pathophysiological roles of the TR4 nuclear receptor: Lessons learned from mice lacking TR4. Mol. Endocrinol..

[91] Arlt A., Schafer H. (2011). Role of the immediate early response 3 (IER3) gene in cellular stress response, inflammation and tumorigenesis. Eur. J. Cell Biol..

[92] Jin H., Jin H., Suh D.S., Kim T.H., Yeom J.H., Lee K., Bae J. (2015). IER3 is a crucial mediator of TAp73beta-induced apoptosis in cervical cancer and confers etoposide sensitivity. Sci. Rep..

[93] Stachel I., Geismann C., Aden K., Deisinger F., Rosenstiel P., Schreiber S., Sebens S., Arlt A., Schäfer H. (2014). Modulation of nuclear factor E2-related factor-2 (Nrf2) activation by the stress response gene immediate early response-3 (IER3) in colonic epithelial cells: A novel mechanism of cellular adaption to inflammatory stress. J. Biol. Chem..

[94] Guzman J.N., Sanchez-Padilla J., Wokosin D., Kondapalli J., Ilijic E., Schumacker P.T., Surmeier D.J. (2010). Oxidant stress evoked by pacemaking in dopaminergic neurons is attenuated by DJ-1. Nature.

[95] Gorgoglione R., Porcelli V., Santoro A., Daddabbo L., Vozza A., Monné M., Di Noia M.A., Palmieri L., Fiermonte G., Palmieri F. (2019). The human uncoupling proteins 5 and 6 (UCP5/SLC25A14 and UCP6/SLC25A30) transport sulfur oxyanions, phosphate and dicarboxylates. Biochim. Biophys. Acta Bioenerg..

[96] Senapedis W.T., Kennedy C.J., Boyle P.M., Silver P.A. (2011). Whole genome siRNA cell-based screen links mitochondria to Akt signaling network through uncoupling of electron transport chain. Mol. Biol. Cell.

[97] Osman C., Merkwirth C., Langer T. (2009). Prohibitins and the functional compartmentalization of mitochondrial membranes. J. Cell Sci..

[98] Sievers C., Billig G., Gottschalk K., Rudel T. (2010). Prohibitins are required for cancer cell proliferation and adhesion. PLoS One.

[99] Anderson C.J., Kahl A., Fruitman H., Qian L., Zhou P., Manfredi G., Iadecola C. (2020). Prohibitin levels regulate OMA1 activity and turnover in neurons. Cell Death Differ.

[100] Wang Z., Cotney J., Shadel G.S. (2007). Human mitochondrial ribosomal protein MRPL12 interacts directly with mitochondrial RNA polymerase to modulate mitochondrial gene expression. J. Biol. Chem..

[101] Serre V., Rozanska A., Beinat M., Chretien D., Boddaert N., Munnich A., Rötig A., Chrzanowska-Lightowlers Z.M. (2013). Mutations in mitochondrial ribosomal protein MRPL12 leads to growth retardation, neurological deterioration and mitochondrial translation deficiency. Biochim. Biophys. Acta.

[102] Zhang Q., Liang Z., Gao Y., Teng M., Niu L. (2017). Differentially expressed mitochondrial genes in breast cancer cells: Potential new targets for anti-cancer therapies. Gene.

[103] Chiche J., Rouleau M., Gounon P., Brahimi-Horn M.C., Pouysségur J., Mazure N.M. (2010). Hypoxic enlarged mitochondria protect cancer cells from apoptotic stimuli. J. Cell Physiol..

[104] Qin Y., Deng Y., Ricketts C.J., Srikantan S., Wang E., Maher E.R., Dahia P.L. (2014). The tumor susceptibility gene TMEM127 is mutated in renal cell carcinomas and modulates endolysosomal function. Hum. Mol. Genet..

[105] Aoyama T., Hata S., Nakao T., Tanigawa Y., Oka C., Kawaichi M. (2009). Cayman ataxia protein caytaxin is transported by kinesin along neurites through binding to kinesin light chains. J. Cell Sci..

[106] Li Y., Tang X., He Q., Yang X., Ren X., Wen X., Zhang J., Wang Y., Liu N., Ma J. (2016). Overexpression of mitochondria mediator gene TRIAP1 by miR-320b loss is associated with progression in nasopharyngeal carcinoma. PLoS Genet..

[107] Andrysik Z., Kim J., Tan A.C., Espinosa J.M. (2013). A genetic screen identifies TCF3/E2A and TRIAP1 as pathway-specific regulators of the cellular response to p53 activation. Cell Rep..

[108] Venco P., Bonora M., Giorgi C., Papaleo E., Iuso A., Prokisch H., Pinton P., Tiranti V. (2015). Mutations of C19orf12, coding for a transmembrane glycine zipper containing mitochondrial protein, cause mis-localization of the protein, inability to respond to oxidative stress and increased mitochondrial Ca(2)(+). Front. Genet..

[109] Hartig M.B., Iuso A., Haack T., Kmiec T., Jurkiewicz E., Heim K., Roeber S., Tarabin V., Dusi S., Krajewska-Walasek M., Jozwiak S., Hempel M., Winkelmann J., Elstner M., Oexle K. (2011). Absence of an orphan mitochondrial protein, c19orf12, causes a distinct clinical subtype of neurodegeneration with brain iron accumulation. Am. J. Hum. Genet..

[110] Mi Y., Shi Z., Li J. (2015). Spata19 is critical for sperm mitochondrial function and male fertility. Mol. Reprod. Dev..

