# Supplementary material for: Disruption of mitochondrial quality control genes promotes caspase-resistant cell survival following apoptotic stimuli
Source: J Biol Chem. 2022 Mar 16;298(4):101835. doi: 10.1016/j.jbc.2022.101835 (PMC9018395; doi:10.1016/j.jbc.2022.101835)
Supplement: Supplemental Figures S1 and S2, Tables S1 and S2 [file mmc1.pdf]

SUPPLEMENTARY DATA

Disruption of Mitochondrial Quality Control Genes Promotes Caspase-Resistant Cell Survival Following Apoptotic Stimuli

Yulia Kushnareva, Vivian Moraes, Julian Suess, Bjoern Peters, Donald D. Newmeyer and Tomomi Kuwana

Table S1. **Mitochondria-annotated genes targeted by siGENOME siRNA pools in the primary screen**. The list indicates gene names, Entrez gene ID and corresponding siRNA catalog numbers.

Table S2. **Secondary screen results summary**. Numbers show assay scores (% of cells) for top candidates in each of the three phenotype categories. Genes in boldface (RMDN3, ATG12, and BNIP3L) were selected for further experiments.

Figure S1. Knockdown efficiencies of selected siRNA pools in Venus-BAX/OMI-mCherry HeLa cells. Cells were reverse- transfected with a non-targeting (nt) and indicated siRNAs at 25-50 nM. Transfection conditions were essentially as described in Methods for screening experiments but scaled up to 6-well plate format to obtain sufficient amounts of cells for western blot analysis. TOM20 was used as loading control.

Figure S2. CRISPR/Cas9-mediated knockout of selected gene candidates. (A) Single guide RNA sequences and CRISPR-mediated gene editing efficiencies in U2OS cells. (**B**) An example of ICE analysis of CRISPR-edited genomic regions shown for RMDN3 knockout cell pool and clone. Other details of the experiments are described in Methods.

Table S1

**AARS**	16	M-011565-01	**ANXA7**	**310**	**M-010760-01**	**BCL2L2**	**599**	**M-004384-02**
**AASS**	10157	M-009247-00	**APBB1**	322	M-011213-03	**BCR**	613	M-003875-05
**ABCA12**	26154	M-008407-01	**APC**	324	M-003869-01	**BDH**	622	M-008643-02
**ABCA4**	24	M-009533-02	**APEX1**	328	M-010237-01	**BECN1**	8678	M-010552-01
**ABCA8**	10351	M-008347-00	**APEX2**	27301	M-013730-00	**BEST1**	7439	M-019825-01
**ABCB10**	23456	M-007300-00	**APOE**	348	M-006470-00	**BID**	637	M-004387-02
**ABCB11**	8647	M-007301-00	**APOO**	79135	M-014318-00	**BIK**	638	M-004388-02
**ABCB4**	5244	M-007302-01	**APOOL**	139322	M-027214-01	**BIRC2**	329	M-004390-02
**ABCB5**	340273	M-007303-01	**AREG**	374	M-017435-00	**BIRC3**	330	M-004099-02
**ABCB6**	10058	M-007304-00	**ARL13B**	200894	M-017365-00	**BIRC4**	331	M-004098-01
**ABCB7**	22	M-007305-00	**ARL2**	402	M-011585-01	**BLOC1S1**	2647	M-012580-00
**ABCB8**	11194	M-007306-01	**ARL2BP**	23568	M-013074-00	**BLOC1S2**	282991	M-018342-00
**ABCC9**	10060	M-007316-01	**ARL6**	84100	M-017298-00	**BMF**	90427	M-004393-04
**ABCD1**	215	M-009605-01	**ARL6IP5**	10550	M-012229-00	**BNIP1**	662	M-011222-01
**ABCD3**	5825	M-009909-01	**ARMCX3**	51566	M-013326-00	**BNIP3**	664	M-004636-01
**ABCE1**	6059	M-008702-01	**ASAH2**	56624	M-005229-00	**BNIP3L**	665	M-011815-01
**ABCF2**	10061	M-009286-00	**ASS**	445	M-010257-03	**BOC**	91653	M-008413-00
**ABCG2**	9429	M-009924-01	**ATAD3A**	55210	M-008191-01	**BOK**	666	M-004394-00
**ABL1**	25	M-003100-02	**ATAD3B**	83858	M-019443-01	**BRI3BP**	140707	M-017153-00
**ACAA2**	10449	M-008773-00	**ATCAY**	85300	M-027139-01	**BRP44**	25874	M-020233-01
**ACACB**	32	M-004759-02	**ATF2**	1386	M-009871-00	**BRP44L**	51660	M-020459-00
**ACAD9**	28976	M-009002-00	**ATG12**	9140	M-010212-02	**C12orf62**	84987	M-015041-00
**ACADL**	33	M-009851-00	**ATG4D**	84971	M-005790-01	**C14ORF112**	51241	M-021166-01
**ACADS**	35	M-010041-02	**ATG5**	9474	M-004374-04	**C14ORF160**	79944	M-008130-01
**ACADVL**	37	M-009392-02	**ATP1A1**	476	M-006111-02	**C14ORF2**	9556	M-019882-01
**ACAT1**	38	M-009408-00	**ATP1B1**	481	M-008381-00	**C14ORF68**	283600	M-007317-01
**ACIN1**	22985	M-014157-01	**ATP2A1**	487	M-006113-00	**C17ORF35**	8834	M-005440-01
**ACSL1**	2180	M-011654-00	**ATP2B2**	491	M-006116-00	**C18orf55**	29090	M-020711-01
**ACSL3**	2181	M-010061-00	**ATP5A1**	498	M-017064-01	**C19ORF12**	83636	M-014731-01
**ACSL4**	2182	M-009364-00	**ATP5B**	506	M-018615-01	**C1orf151**	440574	M-033619-00
**ACSL5**	51703	M-006327-00	**ATP5C1**	509	M-012705-01	**C1orf166**	79594	M-007062-02
**ACSL6**	23305	M-007748-01	**ATP5D**	513	M-017852-01	**C1orf31**	388753	M-027533-01
**ACTB**	60	M-003451-03	**ATP5E**	514	M-012330-00	**C1orf41**	51668	M-010908-01
**ACTC1**	70	M-012015-01	**ATP5F1**	515	M-015956-01	**C1orf78**	55194	M-021255-00
**ACTG1**	71	M-005265-01	**ATP5G1**	516	M-019935-01	**C1QBP**	708	M-011225-00
**ACTN2**	88	M-011196-02	**ATP5G2**	517	M-019936-01	**C1QR1**	22918	M-007815-02
**ADCK4**	79934	M-005305-01	**ATP5G3**	518	M-019494-01	**C20ORF155**	54675	M-017910-00
**ADORA2A**	135	M-005416-02	**ATP5H**	10476	M-012733-02	**C20ORF52**	140823	M-015268-02
**ADRBK1**	156	M-004325-02	**ATP5I**	521	M-019688-01	**C20ORF7**	79133	M-014317-02
**AFG3L2**	10939	M-005781-00	**ATP5J**	522	M-017536-01	**C21ORF2**	755	M-019913-01
**AGER**	177	M-003625-02	**ATP5J2**	9551	M-012690-02	**C22orf29**	79680	M-016338-01
**AGPAT5**	55326	M-008554-02	**ATP5L**	10632	M-017969-01	**C22orf32**	91689	M-016704-00
**AGTR2**	186	M-005429-02	**ATP5O**	539	M-019495-00	**C2ORF18**	54978	M-018201-01
**AHR**	196	M-004990-01	**ATP5S**	27109	M-020544-02	**C2orf33**	56947	M-018261-01
**AK2**	204	M-006812-00	**ATP7A**	538	M-019280-01	**C2orf64**	493753	M-034899-00
**AKAP1**	8165	M-011426-02	**ATPIF1**	93974	M-017220-00	**C3**	718	M-011001-02
**AKAP10**	11216	M-017300-01	**AUH**	549	M-008457-00	**C3ORF1**	51300	M-021164-01
**AKIP**	54998	M-006467-00	**BAD**	572	M-003870-02	**C3orf28**	26355	M-016642-02
**AKR1B10**	57016	M-009691-01	**BAG1**	573	M-003871-02	**C3orf31**	132001	M-016534-01
**AKT2**	208	M-003001-02	**BAG3**	9531	M-011957-01	**C3orf60**	25915	M-027327-01
**AKT3**	10000	M-003002-02	**BAG5**	9529	M-011960-01	**C4ORF14**	84273	M-014851-01
**ALAS2**	212	M-008589-01	**BAK1**	578	M-003305-02	**C4orf35**	85438	M-018644-01
**ALDH1B1**	219	M-008254-00	**BAX**	581	M-003308-03	**C5**	727	M-007819-00
**ALDH3A2**	224	M-009386-03	**BBC3**	27113	M-004380-01	**C6ORF125**	84300	M-021290-01
**ALKBH7**	84266	M-014847-00	**BBOX1**	8424	M-011472-01	**C6ORF168**	84553	M-014910-00
**ALOX12**	239	M-004558-01	**BCAP31**	10134	M-018679-00	**C6ORF49**	29964	M-006979-01
**ALOX5**	240	M-004530-02	**BCDO2**	83875	M-014757-01	**C6ORF66**	29078	M-020684-01
**ALS2CR3**	66008	M-014141-00	**BCKDHB**	594	M-009464-03	**C7ORF23**	79161	M-004156-00
**AMBRA1**	55626	M-029987-01	**BCL2**	596	M-003307-06	**C7ORF27**	221927	M-016785-01
**AMFR**	267	M-006522-01	**BCL2A1**	597	M-003306-01	**C7orf44**	55744	M-020238-01
**AMID**	84883	M-004443-01	**BCL2L1**	598	M-003458-06	**C8orf37**	157657	M-017967-00
**ANKRD15**	23189	M-012879-01	**BCL2L10**	10017	M-004382-01	**C8orf38**	137682	M-016179-01

Table S1

**ANXA1**	301	M-011161-01	**BCL2L11**	10018	M-004383-02	**C9ORF111**	375775	M-010312-01
**ANXA5**	308	M-011209-01	**BCL2L13**	23786	M-020290-00	**C9ORF89**	84270	M-016972-01
**ANXA6**	309	M-011210-01	**BCL2L14**	79370	M-004385-03	**C9ORF90**	203245	M-021314-01
**CA4**	762	M-008775-00	**CLIC4**	25932	M-013553-00	**CYP1B1**	1545	M-008282-00
**CABC1**	56997	M-004939-02	**CLN8**	2055	M-013304-01	**CYP24A1**	1591	M-009269-01
**CACNA2D3**	55799	M-021246-01	**CLPP**	8192	M-005811-01	**CYP27A1**	1593	M-008233-00
**CAMK2A**	815	M-004942-00	**CLPX**	10845	M-008763-00	**CYP27B1**	1594	M-009757-01
**CAMK2G**	818	M-004536-01	**CLRN1**	7401	M-021440-00	**CYP2E1**	1571	M-010134-01
**CAMLG**	819	M-011601-01	**CLU**	1191	M-019513-00	**CYP3A4**	1576	M-008169-00
**CAPN1**	823	M-005799-00	**CNGA1**	1259	M-006158-01	**CYP3A5**	1577	M-009684-00
**CAPS**	828	M-011823-00	**CNGA3**	1261	M-006159-02	**D1S155E**	7812	M-015834-01
**CASP1**	834	M-004401-03	**CNGB1**	1258	M-006160-01	**DACH1**	1602	M-013222-01
**CASP2**	835	M-003465-03	**CNP**	1267	M-018646-01	**DAO**	1610	M-009756-02
**CASP3**	836	M-004307-02	**CNR2**	1269	M-005469-01	**DAOA**	267012	M-015665-01
**CASP6**	839	M-004406-02	**COASY**	80347	M-006751-00	**DAP13**	55967	M-009094-00
**CASP7**	840	M-004407-02	**COCH**	1690	M-011845-00	**DAP3**	7818	M-004416-00
**CASP8**	841	M-003466-05	**COQ10A**	93058	M-018734-01	**DAPK1**	1612	M-004417-03
**CASP9**	842	M-003309-01	**COQ10B**	80219	M-018559-01	**DCI**	1632	M-008794-01
**CASQ1**	844	M-011227-01	**COQ2**	27235	M-018361-01	**DCN**	1634	M-021491-00
**CAT**	847	M-010021-01	**COQ3**	51805	M-010155-01	**DDHD1**	80821	M-021963-01
**CBARA1**	10367	M-012720-01	**COQ4**	51117	M-021015-01	**DDX1**	1653	M-011993-00
**CBFA2T3**	863	M-017195-00	**COQ6**	51004	M-009942-02	**DDX17**	10521	M-013450-01
**CBLB**	868	M-003004-02	**COQ7**	10229	M-017296-00	**DDX18**	8886	M-013451-00
**CCDC109A**	90550	M-015519-00	**COX10**	1352	M-013022-00	**DDX19**	11269	M-013471-01
**CCDC109B**	55013	M-016108-00	**COX11**	1353	M-011837-00	**DDX20**	11218	M-013472-00
**CCDC123**	84902	M-021334-01	**COX15**	1355	M-021442-01	**DDX21**	9188	M-011919-01
**CCDC50**	152137	M-017781-01	**COX18**	285521	M-018117-01	**DDX23**	9416	M-019861-00
**CCDC56**	28958	M-020558-01	**COX4I1**	1327	M-011625-00	**DDX24**	57062	M-010397-01
**CCDC59**	29080	M-020693-01	**COX4I2**	84701	M-013590-00	**DDX31**	64794	M-012931-01
**CCDC90A**	63933	M-010730-01	**COX5A**	9377	M-011940-00	**DDX39**	10212	M-004920-01
**CCDC90B**	60492	M-016774-00	**COX5B**	1329	M-013632-00	**DDX3X**	1654	M-006874-01
**CCNB1**	891	M-003206-02	**COX6A1**	1337	M-011836-00	**DDX41**	51428	M-010394-00
**CCS**	9973	M-008954-01	**COX6B1**	1340	M-013148-00	**DDX42**	11325	M-012393-00
**CD163L1**	283316	M-008024-02	**COX6B2**	125965	M-019413-02	**DDX47**	51202	M-016299-00
**CD34**	947	M-019503-01	**COX6C**	1345	M-013151-01	**DDX48**	9775	M-020762-00
**CD36**	948	M-010206-01	**COX7A1**	1346	M-013152-00	**DDX5**	1655	M-003774-01
**CD68**	968	M-011236-01	**COX7A2**	1347	M-011626-02	**DDX50**	79009	M-004255-00
**CDC2**	983	M-003224-03	**COX7A2L**	9167	M-013179-00	**DDX54**	79039	M-017128-01
**CDH23**	64072	M-013051-01	**COX7B**	1349	M-011627-00	**DDX55**	57696	M-027082-01
**CDK2**	1017	M-003236-04	**COX7B2**	170712	M-015309-01	**DDX56**	54606	M-020410-01
**CDK5**	1020	M-003239-01	**COX7C**	1350	M-013317-00	**DDX58**	23586	M-012511-01
**CDK9**	1025	M-003243-03	**COX8A**	1351	M-011819-01	**DES**	1674	M-011638-02
**CDKN1C**	1028	M-003244-03	**COX8C**	341947	M-019415-01	**DFNB31**	25861	M-026195-01
**CDS1**	1040	M-008680-01	**CPOX**	1371	M-015972-00	**DHDDS**	79947	M-010399-01
**CDS2**	8760	M-009591-01	**CPS1**	1373	M-009275-01	**DHFRL1**	200895	M-009013-03
**CDW92**	23446	M-010708-01	**CPT1A**	1374	M-009749-02	**DHODH**	1723	M-009619-00
**CENTA2**	55803	M-020444-02	**CPT1B**	1375	M-010266-01	**DHRS1**	115817	M-008577-00
**CFH**	3075	M-007920-02	**CPT1C**	126129	M-008824-01	**DHX34**	9704	M-032233-01
**CFLAR**	8837	M-003772-06	**CPT2**	1376	M-008574-01	**DHX38**	9785	M-013428-01
**CHCHD1**	118487	M-027351-00	**CRAT**	1384	M-009524-00	**DIA1**	1727	M-009554-01
**CHCHD3**	54927	M-020803-01	**CRB1**	23418	M-012404-01	**DIABLO**	56616	M-004447-00
**CHCHD4**	131474	M-016042-01	**CRTC1**	23373	M-014026-02	**DIAPH1**	1729	M-010347-02
**CHCHD6**	84303	M-014866-00	**CRTC2**	200186	M-018947-02	**DIP**	23151	M-024634-01
**CHCHD7**	79145	M-014326-01	**CS**	1431	M-009334-01	**DISC1**	27185	M-020321-02
**CHDH**	55349	M-008123-01	**CSNK2A1**	1457	M-003475-03	**DKFZP566**	81889	M-014695-01
**CHPF**	79586	M-017704-00	**CSPG5**	10675	M-020164-01	**DLAT**	1737	M-008490-00
**CHST3**	9469	M-003958-01	**CTSB**	1508	M-004266-03	**DLD**	1738	M-009509-01
**CHUK**	1147	M-003473-02	**CTSD**	1509	M-003649-00	**DLK1**	8788	M-015911-01
**CIAS1**	114548	M-017367-00	**CUTA**	51596	M-020978-01	**DLST**	1743	M-009941-02
**CIG5**	91543	M-015423-01	**CYB5**	1528	M-019621-01	**DMPK**	1760	M-004637-01
**CISD1**	55847	M-020954-01	**CYB5B**	80777	M-014633-01	**DNAJA2**	10294	M-012104-01
**CISD2**	493856	M-032593-00	**CYBB**	1536	M-011021-01	**DNAJA3**	9093	M-017792-00

Table S1

**CKMT1A**	548596	M-034935-01	**CYC1**	1537	M-016697-01	**DNAJC11**	55735	M-021205-01
**CKMT1B**	1159	M-006708-01	**CYCS**	54205	M-017355-00	**DNAJC15**	29103	M-020286-01
**CKMT2**	1160	M-006709-01	**CYP11A1**	1583	M-008329-02	**DNAJC19**	131118	M-016024-00
**CLCN3**	1182	M-006151-02	**CYP11B1**	1584	M-008635-02	**DNCL1**	8655	M-005281-02
**CLDN1**	9076	M-017369-01	**CYP11B2**	1585	M-009021-01	**DNLZ**	728489	M-184311-01
**CLIC1**	1192	M-009530-00	**CYP1A1**	1543	M-004790-02	**DNM1L**	10059	M-012092-01
**DNMT1**	1786	M-004605-01	**FUNDC1**	139341	M-018480-01	**HIGD2A**	192286	M-016491-01
**DSG2**	1829	M-011645-01	**FUNDC2**	65991	M-006628-01	**HIP1**	3092	M-005001-01
**DUSP1**	1843	M-003484-02	**FXC1**	26515	M-018242-02	**HK1**	3098	M-006820-01
**DUSP21**	63904	M-007893-01	**GABARAPL1**	23710	M-014715-01	**HK2**	3099	M-006735-01
**DYNC1H1**	1778	M-006828-02	**GABRA5**	2558	M-006166-00	**HK3**	3101	M-006736-00
**DYNLL2**	140735	M-006493-01	**GATA1**	2623	M-009610-00	**HMGB1**	3146	M-018981-01
**DYNLT1**	6993	M-019964-00	**GATM**	2628	M-008900-01	**HMGCL**	3155	M-019290-01
**E2F1**	1869	M-003259-01	**GCDH**	2639	M-004039-01	**HMGCS1**	3157	M-009808-01
**ECGF1**	1890	M-009281-01	**GCH1**	2643	M-010328-03	**HMGCS2**	3158	M-010179-01
**ECH1**	1891	M-004035-01	**GDAP1**	54332	M-021225-01	**HMOX1**	3162	M-006372-02
**ECHS1**	1892	M-010343-00	**GDF5**	8200	M-012271-02	**HNRPM**	4670	M-013452-01
**EFEMP1**	2202	M-011855-01	**GDNF**	2668	M-011040-01	**HRAS**	3265	M-004142-00
**EFHA2**	286097	M-018623-00	**GFAP**	2670	M-011667-00	**HSA9947**	23400	M-008601-01
**EFHD1**	80303	M-010673-00	**GFER**	2671	M-012041-01	**HSCB**	150274	M-017718-01
**EGFL11**	346007	M-024856-00	**GGA2**	23062	M-012908-01	**HSD17B8**	7923	M-008141-02
**EGFR**	1956	M-003114-03	**GGT1**	2678	M-005884-01	**HSD3B1**	3283	M-008972-02
**EI24**	9538	M-019879-01	**GHITM**	27069	M-020534-01	**HSD3B2**	3284	M-012542-01
**EIF2S1**	1965	M-015389-01	**GIMAP5**	55340	M-013342-01	**HSP90B1**	7184	M-006417-02
**EIF3S10**	8661	M-019534-01	**GJA1**	2697	M-011042-01	**HSPA1A**	3303	M-005168-01
**EIF4G1**	1981	M-019474-01	**GJB2**	2706	M-019285-01	**HSPA1B**	3304	M-003501-03
**EIF4G2**	1982	M-011263-01	**GJB3**	2707	M-019948-02	**HSPA4**	3308	M-012636-02
**ELA2**	1991	M-005861-02	**GJB6**	10804	M-019916-01	**HSPA5**	3309	M-008198-02
**ENDOGL1**	9941	M-008572-01	**GK**	2710	M-006727-00	**HSPA9B**	3313	M-004750-03
**ENOSF1**	55556	M-020894-00	**GK2**	2712	M-015091-01	**HSPB1**	3315	M-005269-01
**ENOX1**	55068	M-021077-00	**GLRX**	2745	M-012634-01	**HSPB8**	26353	M-005006-00
**EPHA4**	2043	M-003118-02	**GLRX2**	51022	M-021332-01	**HSPCA**	3320	M-005186-02
**EPHB6**	2051	M-003125-02	**GLTSCR2**	29997	M-006464-01	**HSPD1**	3329	M-010600-02
**EPO**	2056	M-020204-01	**GMIP**	51291	M-021160-01	**HSPE1**	3336	M-019649-00
**ERAL1**	26284	M-012709-01	**GNA12**	2768	M-008435-00	**IAPP**	3375	M-007790-01
**ERBB4**	2066	M-003128-03	**GNB2L1**	10399	M-006876-01	**IBRDC2**	255488	M-025119-01
**ERCC6**	2074	M-004888-01	**GOLPH3**	64083	M-006414-00	**ICT1**	3396	M-010517-00
**ETFA**	2108	M-011029-01	**GOT2**	2806	M-011674-02	**IDE**	3416	M-005899-03
**ETFB**	2109	M-010494-01	**GPAM**	57678	M-009946-01	**IDH2**	3418	M-004013-00
**ETFDH**	2110	M-008127-02	**GPD2**	2820	M-009843-02	**IER3**	8870	M-011547-01
**EYA2**	2139	M-017233-01	**GPR30**	2852	M-005563-02	**IF**	3426	M-005900-02
**F2R**	2149	M-005094-01	**GPR81**	27198	M-005601-02	**IFI27**	3429	M-006465-02
**FAM36A**	116228	M-021429-01	**GPX4**	2879	M-011676-01	**IFI6**	2537	M-003672-02
**FAM82C**	55177	M-020973-00	**GRHL2**	79977	M-014515-01	**IFNA2**	3440	M-013809-01
**FARSLB**	10056	M-015414-01	**GRP58**	2923	M-003674-01	**IFNB1**	3456	M-019656-01
**FASLG**	356	M-011130-00	**GRPEL2**	134266	M-016191-01	**IGF1R**	3480	M-003012-05
**FATE1**	89885	M-015068-01	**GSK3A**	2931	M-003009-01	**IGF2**	3481	M-004093-01
**FCGR2B**	2213	M-015823-01	**GSK3B**	2932	M-003010-03	**IKBKB**	3551	M-003503-03
**FCGR3B**	2215	M-019374-01	**GSN**	2934	M-007775-03	**IKBKE**	9641	M-003723-02
**FECH**	2235	M-011036-01	**GSR**	2936	M-009647-01	**IL7**	3574	M-007995-01
**FEN1**	2237	M-010344-01	**GSTA4**	2941	M-011289-00	**IMMP2L**	83943	M-005902-02
**FGFBP1**	9982	M-019910-00	**GSTK1**	373156	M-020958-01	**IMMT**	10989	M-019832-01
**FGR**	2268	M-003135-03	**GTPBP5**	26164	M-013036-01	**IMPG2**	50939	M-020890-01
**FHIT**	2272	M-004952-02	**GUCA1B**	2979	M-015131-01	**INS**	3630	M-011058-01
**FIS1**	51024	M-020907-02	**GUF1**	60558	M-021270-01	**INSIG2**	51141	M-021039-00
**FKBP5**	2289	M-004224-01	**GZMB**	3002	M-005889-02	**IPLA2gamma**	50640	M-010284-01
**FKBP8**	23770	M-009673-02	**GZMH**	2999	M-005890-02	**IRF1**	3659	M-011704-01
**FKSG24**	84769	M-014959-02	**HAAO**	23498	M-008666-00	**IRS1**	3667	M-003015-01
**FKTN**	2218	M-012313-01	**HADH2**	3028	M-009390-01	**ITGA11**	22801	M-008000-02
**FLCN**	201163	M-009998-01	**HADHA**	3030	M-009470-01	**ITGA2**	3673	M-004566-02
**FLJ12592**	84129	M-008129-01	**HADHB**	3032	M-008280-01	**ITPR3**	3710	M-006209-02
**FLJ14466**	84876	M-014998-01	**HADHSC**	3033	M-008298-01	**ITSN1**	6453	M-008365-01

Table S1

**FLJ25059**	196294	M-005877-01	**HAX1**	10456	M-012168-01	**JTB**	10899	M-010567-01
**FLJ30473**	150209	M-008134-00	**HBB**	3043	M-011047-01	**KARS**	3735	M-012114-00
**FLVCR**	28982	M-020584-01	**HCCS**	3052	M-009226-01	**KCNA3**	3738	M-006213-01
**FMR1**	2332	M-019631-00	**HD**	3064	M-003737-02	**KCNA5**	3741	M-006215-01
**FNDC1**	84624	M-024901-01	**HDAC6**	10013	M-003499-00	**KCNJ10**	3766	M-006240-01
**FOXO3A**	2309	M-003007-02	**HEBP2**	23593	M-020612-00	**KCNK3**	3777	M-006262-02
**FOXRED1**	55572	M-008137-01	**HERC2**	8924	M-007180-02	**KCNK9**	51305	M-004891-01
**FPGS**	2356	M-016472-01	**HGF**	3082	M-006650-01	**KCNN3**	3782	M-006270-02
**FTH1**	2495	M-019634-02	**HIGD1A**	25994	M-020242-01	**KCNN4**	3783	M-004461-01
**KCNQ4**	9132	M-006274-00	**MCART1**	92014	M-007358-01	**MRPL41**	64975	M-013572-00
**KDR**	3791	M-003148-01	**MCART2**	147407	M-031039-01	**MRPL42**	28977	M-017553-00
**KIAA0174**	9798	M-020977-00	**MCART6**	401612	M-031566-02	**MRPL43**	84545	M-019126-00
**KIAA0446**	9673	M-007339-01	**MCCC1**	56922	M-009429-02	**MRPL44**	65080	M-012858-00
**KIAA0774**	23281	M-006850-01	**MCL1**	4170	M-004501-08	**MRPL45**	84311	M-019256-02
**KIAA0831**	22863	M-020438-01	**MDH2**	4191	M-008439-00	**MRPL46**	26589	M-017562-00
**KIF14**	9928	M-003319-00	**ME2**	4200	M-009461-01	**MRPL47**	57129	M-013186-01
**KIF1B**	23095	M-009317-01	**MECP2**	4204	M-013094-02	**MRPL48**	51642	M-017512-02
**KIF5B**	3799	M-008867-00	**MEF2A**	4205	M-009362-00	**MRPL49**	740	M-017507-00
**KLHL7**	55975	M-015574-00	**MEF2C**	4208	M-009455-00	**MRPL50**	54534	M-013373-02
**KMO**	8564	M-009897-01	**MEF2D**	4209	M-009884-00	**MRPL51**	51258	M-013563-00
**KRT18**	3875	M-010604-02	**MERTK**	10461	M-003155-02	**MRPL52**	122704	M-019012-01
**KRT6A**	3853	M-012116-01	**MFN1**	55669	M-010670-01	**MRPL53**	116540	M-012941-00
**KRT8**	3856	M-019658-01	**MFN2**	9927	M-012961-00	**MRPL54**	116541	M-017497-01
**KUB3**	91419	M-015098-01	**MFTC**	81034	M-007356-00	**MRPL55**	128308	M-019054-02
**LAMB2**	3913	M-013310-01	**MGC4767**	84274	M-009755-01	**MRPL9**	65005	M-013498-02
**LAMC3**	10319	M-012173-01	**MGC5352**	192111	M-015919-02	**MRPS10**	55173	M-013345-00
**LASS6**	253782	M-032207-00	**MGEA5**	10724	M-012805-01	**MRPS11**	64963	M-013623-00
**LCK**	3932	M-003151-02	**MGST1**	4257	M-009248-00	**MRPS12**	6183	M-011382-01
**LDB3**	11155	M-026288-02	**MIPEP**	4285	M-005949-02	**MRPS14**	63931	M-013050-00
**LDHA**	3939	M-008201-01	**MJD**	4287	M-012013-01	**MRPS15**	64960	M-013609-00
**LDHB**	3945	M-009779-01	**MKKS**	8195	M-013300-00	**MRPS16**	51021	M-013130-01
**LDHD**	197257	M-008761-01	**MK-STYX**	51657	M-008031-01	**MRPS17**	51373	M-013215-00
**LETM1**	3954	M-019549-00	**MLXIP**	22877	M-008976-01	**MRPS18A**	55168	M-007008-00
**LETM2**	137994	M-015974-01	**MMAA**	166785	M-016320-01	**MRPS18B**	28973	M-013043-00
**LETMD1**	25875	M-016920-01	**MMP1**	4312	M-005951-01	**MRPS18C**	51023	M-013131-00
**LGALS3**	3958	M-010606-02	**MOAP1**	64112	M-004430-02	**MRPS21**	54460	M-013388-00
**LHFPL5**	222662	M-018911-01	**MOSC1**	64757	M-019358-00	**MRPS22**	56945	M-013219-00
**LMNA**	4000	M-004978-01	**MOSC2**	54996	M-018689-00	**MRPS23**	51649	M-012973-01
**LOC150763**	150763	M-010302-01	**MPST**	4357	M-010119-01	**MRPS24**	64951	M-013534-02
**LOC153328**	153328	M-007347-02	**MPV17**	4358	M-017720-01	**MRPS25**	64432	M-010142-00
**LOC201164**	201164	M-017858-00	**MPV17L**	255027	M-018370-00	**MRPS26**	64949	M-013546-00
**LOC203427**	203427	M-007349-01	**MRC1**	4360	M-011730-01	**MRPS27**	23107	M-012903-00
**LOC283130**	283130	M-007351-01	**M-RIP**	23164	M-014102-01	**MRPS28**	28957	M-013492-00
**LOC790955**	790955	M-184603-01	**MRPL1**	65008	M-017264-01	**MRPS30**	10884	M-013207-02
**LPIN1**	23175	M-017427-01	**MRPL10**	124995	M-017394-00	**MRPS31**	10240	M-012111-00
**LPL**	4023	M-008970-01	**MRPL11**	65003	M-013123-00	**MRPS33**	51650	M-012940-00
**LRAT**	9227	M-010272-01	**MRPL12**	6182	M-017517-00	**MRPS34**	65993	M-012884-00
**LRPPRC**	10128	M-018773-00	**MRPL13**	28998	M-013557-00	**MRPS35**	60488	M-013071-00
**LRRC51**	220074	M-016302-01	**MRPL14**	64928	M-013550-01	**MRPS36**	92259	M-019052-01
**LRRC59**	55379	M-010669-00	**MRPL15**	29088	M-013052-00	**MRPS5**	64969	M-019247-00
**LRRK2**	120892	M-006323-02	**MRPL16**	54948	M-013354-00	**MRPS6**	64968	M-019243-00
**LYN**	4067	M-003153-04	**MRPL17**	63875	M-013049-00	**MRPS7**	51081	M-013580-00
**LYRM7**	90624	M-018887-01	**MRPL18**	29074	M-017251-00	**MRPS9**	64965	M-019184-00
**MAG**	4099	M-011722-00	**MRPL19**	9801	M-013418-01	**MRS2L**	57380	M-020748-02
**MAGMAS**	51025	M-015261-00	**MRPL2**	51069	M-017261-00	**MSRA**	4482	M-012464-00
**MAOA**	4128	M-009369-01	**MRPL20**	55052	M-017564-00	**MSTO1**	55154	M-021170-00
**MAOB**	4129	M-010183-03	**MRPL21**	219927	M-019072-02	**MT2A**	4502	M-018338-00
**MAP1A**	4130	M-013482-01	**MRPL22**	29093	M-017259-01	**MTCH1**	23787	M-007370-01
**MAP1LC3A**	84557	M-013579-00	**MRPL23**	6150	M-013124-00	**MTCH2**	23788	M-007371-00
**MAP1LC3B**	81631	M-012846-01	**MRPL24**	79590	M-017442-00	**MTFR1**	9650	M-019432-00
**MAP2K1**	5604	M-003571-01	**MRPL27**	51264	M-013182-01	**MTG1**	92170	M-015522-01
**MAP2K2**	5605	M-003573-03	**MRPL28**	10573	M-017627-00	**MTHFD1L**	25902	M-009949-01

Table S1

**MAP2K3**	5606	M-003509-03	**MRPL3**	11222	M-012372-00	**MTHFD2L**	441024	M-032402-01
**MAP3K12**	7786	M-003312-02	**MRPL30**	51263	M-013181-01	**MTNR1A**	4543	M-005669-02
**MAPK12**	6300	M-003590-03	**MRPL32**	64983	M-013512-01	**MTP18**	51537	M-021196-01
**MAPK3**	5595	M-003592-03	**MRPL33**	9553	M-017401-01	**MTRF1**	9617	M-019211-00
**MAPK8**	5599	M-003514-04	**MRPL34**	64981	M-012885-00	**MTUS1**	57509	M-006848-00
**MAPK8IP1**	9479	M-003595-00	**MRPL35**	51318	M-013206-01	**MTX1**	4580	M-019667-02
**MAPKAP1**	79109	M-014315-02	**MRPL36**	64979	M-013574-00	**MTX2**	10651	M-020087-01
**MAPRE1**	22919	M-006824-00	**MRPL37**	51253	M-017460-00	**MTX3**	345778	M-024528-01
**MARCH5**	54708	M-007001-01	**MRPL38**	64978	M-013573-00	**MUC1**	4582	M-004019-02
**MARK2**	2011	M-004260-02	**MRPL39**	54148	M-008542-01	**MULK**	55750	M-007256-02
**MARVELD2**	153562	M-017054-02	**MRPL4**	51073	M-017470-00	**MYH11**	4629	M-011737-01
**MB**	4151	M-012057-01	**MRPL40**	64976	M-017608-00	**MYH14**	79784	M-027149-01
**MYH6**	4624	M-012645-01	**NNT**	23530	M-009809-01	**PID1**	55022	M-018934-01
**MYH9**	4627	M-007668-01	**NOS1**	4842	M-009496-01	**PIK3CA**	5290	M-003018-03
**MYLK**	4638	M-005351-05	**NOTCH1**	4851	M-007771-02	**PIK3CG**	5294	M-005274-02
**MYO19**	80179	M-017137-02	**NOTCH3**	4854	M-011093-01	**PIK4CB**	5298	M-006777-03
**MYO1A**	4640	M-008765-00	**NOX1**	27035	M-010193-01	**PIM1**	5292	M-003923-00
**MYO6**	4646	M-006355-00	**NOX4**	50507	M-010194-00	**PINK1**	65018	M-004030-02
**MYO7A**	4647	M-019330-01	**NPC1**	4864	M-008047-01	**PISD**	23761	M-009548-00
**MYOC**	4653	M-011089-02	**NPTX1**	4884	M-011343-01	**PLA2G2A**	5320	M-009901-03
**NAT8L**	339983	M-009115-00	**NR2C2**	7182	M-003418-02	**PLA2G4A**	5321	M-009886-01
**NBL1**	4681	M-006540-01	**NR4A1**	3164	M-003426-03	**PLA2G6**	8398	M-009085-04
**NCF1**	653361	M-180696-00	**NRG1**	3084	M-004608-02	**PLA2G7**	7941	M-004903-01
**NCOA7**	135112	M-018862-00	**NTN1**	9423	M-011946-01	**PLAA**	9373	M-016215-01
**NDE1**	54820	M-020625-00	**NTRK1**	4914	M-003159-02	**PLAUR**	5329	M-006388-01
**NDUFA1**	4694	M-011881-01	**NUBPL**	80224	M-021287-01	**PLB1**	151056	M-008684-00
**NDUFA10**	4705	M-006752-00	**NUP93**	9688	M-020767-00	**PLEKHF1**	79156	M-018423-01
**NDUFA11**	126328	M-018508-00	**OCIAD2**	132299	M-015633-01	**PLEKHF2**	79666	M-018407-00
**NDUFA12L**	91942	M-017758-01	**OGDH**	4967	M-009679-02	**PLG**	5340	M-006001-02
**NDUFA13**	51079	M-016921-01	**OGG1**	4968	M-005147-03	**PLN**	5350	M-011754-00
**NDUFA2**	4695	M-018869-01	**OLA1**	29789	M-015680-01	**PLOD2**	5352	M-004285-01
**NDUFA4**	4697	M-019200-00	**OMA1**	115209	M-008662-03	**PLSCR3**	57048	M-010255-00
**NDUFA5**	4698	M-012000-00	**OPA1**	4976	M-005273-00	**PMAIP1**	5366	M-005275-03
**NDUFA6**	4700	M-015716-01	**OPA3**	80207	M-014595-01	**PMPCA**	23203	M-008734-01
**NDUFA7**	4701	M-012693-01	**OPRD1**	4985	M-005683-01	**PMPCB**	9512	M-004747-01
**NDUFA8**	4702	M-012496-00	**OPTN**	10133	M-016269-02	**PNKP**	11284	M-006783-02
**NDUFA9**	4704	M-016044-02	**OSAP**	84709	M-010682-02	**PNPLA2**	57104	M-009003-01
**NDUFAB1**	4706	M-019897-01	**OTC**	5009	M-009291-02	**PNPLA3**	80339	M-009564-01
**NDUFAF1**	51103	M-021003-02	**OTOA**	146183	M-016394-01	**PNPT1**	87178	M-019454-00
**NDUFB1**	4707	M-017848-02	**OTOF**	9381	M-011942-00	**POLG**	5428	M-012649-00
**NDUFB10**	4716	M-012675-01	**OXA1L**	5018	M-012696-00	**POLR2B**	5431	M-011187-00
**NDUFB11**	54539	M-016098-02	**P2RX5**	5026	M-006286-04	**PON2**	5445	M-009676-01
**NDUFB2**	4708	M-019202-02	**P2RX7**	5027	M-003728-01	**PON3**	5446	M-009675-02
**NDUFB3**	4709	M-019604-01	**PAEP**	5047	M-010027-02	**PP591**	80308	M-008629-00
**NDUFB4**	4710	M-032508-00	**PAK1**	5058	M-003521-04	**PPARA**	5465	M-003434-01
**NDUFB5**	4711	M-019209-02	**PANK2**	80025	M-003797-03	**PPIF**	10105	M-009708-00
**NDUFB6**	4712	M-017210-00	**PAPD1**	55149	M-016486-00	**PPOX**	5498	M-008383-01
**NDUFB7**	4713	M-017213-01	**PARG**	8505	M-011488-02	**PPP1CA**	5499	M-008927-01
**NDUFB8**	4714	M-019898-01	**PARK2**	5071	M-003603-00	**PPP1R15A**	23645	M-004442-01
**NDUFB9**	4715	M-019899-01	**PARK7**	11315	M-005984-00	**PPP2R2B**	5521	M-003022-02
**NDUFC1**	4717	M-019601-02	**PARL**	55486	M-021387-01	**PPP3CC**	5533	M-010005-00
**NDUFC2**	4718	M-015319-01	**PARP1**	142	M-006656-01	**PPP3R1**	5534	M-009869-02
**NDUFS1**	4719	M-019069-00	**PARP4**	143	M-007244-03	**PRCD**	768206	M-183754-01
**NDUFS2**	4720	M-015770-01	**PBEF1**	10135	M-004581-01	**PRDX3**	10935	M-010355-00
**NDUFS3**	4722	M-019815-01	**PC**	5091	M-008950-02	**PRDX5**	25824	M-019102-00
**NDUFS4**	4724	M-019602-00	**PCDH15**	65217	M-013654-01	**PRELID1**	27166	M-017650-01
**NDUFS5**	4725	M-019816-00	**PDCD5**	9141	M-004439-01	**PREP**	5550	M-006006-01
**NDUFS6**	4726	M-019817-01	**PDCD8**	9131	M-011912-00	**PRKAA1**	5562	M-005027-02
**NDUFS7**	374291	M-031021-00	**PDE2A**	5138	M-007644-00	**PRKAB1**	5564	M-007675-00
**NDUFS8**	4728	M-019600-00	**PDE6A**	5145	M-007651-00	**PRKACA**	5566	M-004649-01
**NDUFV1**	4723	M-016266-00	**PDE6B**	5158	M-007652-01	**PRKAG3**	53632	M-009859-01
**NDUFV2**	4729	M-012589-01	**PDE6G**	5148	M-007655-01	**PRKCA**	5578	M-003523-03

Table S1

**NDUFV3**	4731	M-016362-01	**PDGFB**	5155	M-011749-00	**PRKCD**	5580	M-003524-01
**NF1**	4763	M-003916-03	**PDGFRA**	5156	M-003162-04	**PRKCE**	5581	M-004653-02
**NFATC1**	4772	M-003605-03	**PDK4**	5166	M-019425-02	**PRKCM**	5587	M-005028-02
**NFE2L2**	4780	M-003755-02	**PDZK1**	5174	M-010615-03	**PRKCQ**	5588	M-003525-01
**NFKBIA**	4792	M-004765-00	**PEA15**	8682	M-010553-01	**PRKCZ**	5590	M-003526-04
**NFS1**	9054	M-011564-00	**PECI**	10455	M-009804-00	**PRKD2**	25865	M-004197-02
**NGFR**	4804	M-009340-02	**PEMT**	10400	M-010392-00	**PRKG1**	5592	M-004658-04
**NHEDC2**	133308	M-007345-00	**PEX13**	5194	M-012591-02	**PRKR**	5610	M-003527-00
**NIPSNAP1**	8508	M-011489-00	**PEX3**	8504	M-019544-00	**PRNP**	5621	M-011101-01
**NLN**	57486	M-005977-01	**PFKP**	5214	M-010253-01	**PRODH**	5625	M-009543-00
**NLRX1**	79671	M-012926-01	**PGR**	5241	M-003433-01	**PRODH2**	58510	M-013098-01
**NME1**	4830	M-006821-01	**PGS1**	9489	M-009483-01	**PROM1**	8842	M-010630-01
**NME2**	4831	M-005102-02	**PHB**	5245	M-010530-00	**PRPF6**	24148	M-012821-01
**NME4**	4833	M-006494-00	**PHLDA2**	7262	M-011411-01	**PRPF8**	10594	M-012252-02
**NMT1**	4836	M-004316-00	**PI4KII**	55361	M-006770-02	**PRSS11**	5654	M-006009-02
**PRSS15**	9361	M-003979-00	**SAC**	55811	M-006353-01	**SLC25A29**	123096	M-007318-01
**PRSS25**	27429	M-006014-04	**SAMM50**	25813	M-017871-00	**SLC25A3**	5250	M-007484-00
**PSEN1**	5663	M-004998-01	**SARM1**	23098	M-008076-01	**SLC25A30**	253512	M-007350-01
**PSEN2**	5664	M-006018-02	**SCN1A**	6323	M-006297-02	**SLC25A31**	83447	M-007322-01
**PTCD3**	55037	M-016957-00	**SCN5A**	6331	M-006500-03	**SLC25A33**	84275	M-007366-01
**PTEN**	5728	M-003023-02	**SCO1**	6341	M-011892-01	**SLC25A34**	284723	M-032041-02
**PTGIS**	5740	M-004691-02	**SCO2**	9997	M-011987-01	**SLC25A35**	399512	M-031743-01
**PTK2**	5747	M-003164-02	**SDHA**	6389	M-009398-02	**SLC25A36**	55186	M-007327-02
**PTK2B**	2185	M-003165-03	**SDHB**	6390	M-011773-02	**SLC25A37**	51312	M-007369-01
**PTMA**	5757	M-005207-01	**SDHC**	6391	M-011385-01	**SLC25A38**	54977	M-007331-01
**PTPMT1**	114971	M-029988-02	**SDHD**	6392	M-006305-00	**SLC25A39**	51629	M-007319-00
**PTPN1**	5770	M-003529-04	**SEMA4A**	64218	M-015686-00	**SLC25A4**	291	M-007485-02
**PTPN11**	5781	M-003947-01	**SERAC1**	84947	M-015026-00	**SLC25A40**	55972	M-007354-01
**PTPRC**	5788	M-008067-01	**SERPINA1**	5265	M-008847-01	**SLC25A41**	284427	M-007363-01
**PTRH2**	51651	M-007271-01	**SET**	6418	M-019586-01	**SLC25A42**	284439	M-007361-01
**PXN**	5829	M-005163-00	**SFN**	2810	M-005180-00	**SLC25A46**	91137	M-007353-00
**PYCR1**	5831	M-012349-00	**SFRP4**	6424	M-011388-01	**SLC25A5**	292	M-007486-03
**PYCS**	5832	M-006785-01	**SFXN1**	94081	M-010686-00	**SLC25A6**	293	M-007487-01
**RAB11A**	8766	M-004726-02	**SFXN2**	118980	M-018547-00	**SLC26A4**	5172	M-007493-01
**RAB11FIP1**	80223	M-015968-01	**SFXN3**	81855	M-018729-02	**SLC27A3**	11000	M-007499-01
**RAB11FIP5**	26056	M-004298-01	**SFXN4**	119559	M-018237-01	**SLC29A1**	2030	M-003709-01
**RAB32**	10981	M-009920-02	**SFXN5**	94097	M-016367-01	**SLC2A1**	6513	M-007509-01
**RAC1**	5879	M-003560-06	**SGCD**	6444	M-017292-00	**SLC2A4**	6517	M-007517-02
**RAC2**	5880	M-007741-01	**SGK**	6446	M-003027-05	**SLC35B3**	51000	M-007544-00
**RAF1**	5894	M-003601-02	**SH120**	51463	M-005725-01	**SLC35C1**	55343	M-010693-00
**RALA**	5898	M-009235-00	**SH3BP5**	9467	M-019869-01	**SLC37A4**	2542	M-007557-01
**RALBP1**	10928	M-009266-00	**SH3GLB1**	51100	M-017086-01	**SLC38A4**	55089	M-007561-01
**RAP1B**	5908	M-010364-03	**SHC1**	6464	M-018841-02	**SLC3A1**	6519	M-007575-00
**RDH12**	145226	M-008319-00	**SHH**	6469	M-006036-02	**SLC5A1**	6523	M-007589-01
**RDH13**	112724	M-008628-00	**SHMT2**	6472	M-004906-01	**SLC6A1**	6529	M-007597-01
**RDS**	5961	M-011102-01	**SIGLEC8**	27181	M-012843-01	**SLC8A2**	6543	M-007621-01
**RDX**	5962	M-011762-02	**SIRT1**	23411	M-003540-01	**SLC8A3**	6547	M-007622-02
**REA**	11331	M-018703-00	**SIRT3**	23410	M-004827-01	**SLC9A1**	6548	M-005277-00
**RECQL4**	9401	M-010559-01	**SIVA**	10572	M-012262-02	**SLC9A3R1**	9368	M-012688-01
**REEP1**	65055	M-014235-00	**SLC11A2**	4891	M-007381-01	**SLCO1A2**	6579	M-007439-00
**RELA**	5970	M-003533-02	**SLC16A7**	9194	M-007409-01	**SMAD5**	4090	M-015791-00
**REN**	5972	M-006026-01	**SLC17A8**	246213	M-007418-00	**SMCP**	4184	M-017503-00
**REP15**	387849	M-030132-01	**SLC19A1**	6573	M-007422-01	**SMCR7**	125170	M-017272-00
**RET**	5979	M-003170-02	**SLC1A3**	6507	M-007427-00	**SMCR7L**	54471	M-015938-00
**RFFL**	117584	M-007120-03	**SLC1A4**	6509	M-007428-01	**SMPD1**	6609	M-006676-01
**RGR**	5995	M-005721-02	**SLC22A3**	6581	M-007454-00	**SMPD4**	55627	M-020681-02
**RHO**	6010	M-005722-00	**SLC22A7**	10864	M-007458-01	**SMURF1**	57154	M-007191-01
**RHOA**	387	M-003860-03	**SLC23A2**	9962	M-007462-01	**SNN**	8303	M-018536-01
**RHOT1**	55288	M-010365-01	**SLC24A6**	80024	M-007332-00	**SNPH**	9751	M-020417-00
**RHOT2**	89941	M-008340-01	**SLC25A1**	6576	M-007468-01	**SNTA1**	6640	M-011777-00
**RIMS3**	9783	M-020891-00	**SLC25A10**	1468	M-007469-00	**SNTG1**	54212	M-021231-01
**RIPK1**	8737	M-004445-02	**SLC25A11**	8402	M-007470-00	**SOD2**	6648	M-009784-02

Table S1

**RLBP1L1**	157807	M-018542-01	**SLC25A12**	8604	M-007471-01	**SORD**	6652	M-008323-02
**RNF185**	91445	M-007107-01	**SLC25A13**	10165	M-007472-01	**SOX10**	6663	M-017192-00
**RNF34**	80196	M-007072-00	**SLC25A14**	9016	M-007473-00	**SPATA18**	132671	M-016038-01
**RNF5**	6048	M-006558-02	**SLC25A15**	10166	M-007474-02	**SPATA19**	219938	M-017842-00
**ROBO3**	64221	M-026504-00	**SLC25A16**	8034	M-007475-00	**SPG20**	23111	M-015681-02
**ROM1**	6094	M-017459-00	**SLC25A17**	10478	M-007476-01	**SPG7**	6687	M-006039-02
**RP2**	6102	M-012350-01	**SLC25A18**	83733	M-007477-01	**SPIN1**	83985	M-003987-03
**RPE65**	6121	M-008199-01	**SLC25A19**	60386	M-007478-02	**SPTLC1**	10558	M-006673-02
**RPS3**	6188	M-013607-01	**SLC25A2**	83884	M-007479-01	**SQRDL**	58472	M-008271-01
**RPS6KB1**	6198	M-003616-03	**SLC25A20**	788	M-007480-03	**SRC**	6714	M-003175-03
**RPS6KC1**	26750	M-005371-02	**SLC25A21**	89874	M-007481-01	**STAR**	6770	M-019369-01
**RUFY2**	55680	M-007016-01	**SLC25A22**	79751	M-007482-01	**STARD13**	90627	M-010256-00
**RUNX1**	861	M-003926-02	**SLC25A23**	79085	M-007360-01	**STARD3**	10948	M-017665-00
**RUNX2**	860	M-012665-01	**SLC25A24**	29957	M-007325-01	**STARD7**	56910	M-017289-01
**RXRA**	6256	M-003443-02	**SLC25A25**	114789	M-007355-01	**STAT2**	6773	M-012064-00
**S100A8**	6279	M-011770-02	**SLC25A26**	115286	M-007343-01	**STAT5A**	6776	M-005169-02
**S100A9**	6280	M-011384-02	**SLC25A27**	9481	M-007483-01	**STAT5B**	6777	M-010539-02
**S100B**	6285	M-012259-02	**SLC25A28**	81894	M-007368-01	**STIM1**	6786	M-011785-00
**STK32A**	202374	M-004634-01	**TP73**	7161	M-003331-01	**WASF1**	8936	M-011557-01
**STOM**	2040	M-016971-00	**TPM1**	7168	M-017837-01	**WFS1**	7466	M-009740-01
**STOML2**	30968	M-020518-00	**TPRT**	23590	M-008464-01	**WWOX**	51741	M-003961-03
**STOX1**	219736	M-017070-01	**TPT1**	7178	M-004559-03	**XBP1**	7494	M-009552-02
**STX17**	55014	M-020965-01	**TRAF2**	7186	M-005198-00	**YBX1**	4904	M-010213-03
**SUCLG1**	8802	M-008677-01	**TRAK1**	22906	M-020331-02	**YIPF1**	54432	M-016906-00
**SUCLG2**	8801	M-008918-01	**TRAP1**	10131	M-010104-01	**YME1L1**	10730	M-006103-01
**SURF1**	6834	M-011786-01	**TREM1**	54210	M-017974-00	**YRDC**	79693	M-018139-01
**SYNJ2BP**	55333	M-021176-01	**TRIAP1**	51499	M-020809-00	**YWHAB**	7529	M-008766-02
**TARDBP**	23435	M-012394-01	**TRPC3**	7222	M-006509-01	**YWHAE**	7531	M-017302-02
**TARP**	445347	M-032367-02	**TRPM2**	7226	M-004193-02	**YWHAG**	7532	M-008844-00
**TAZ**	6901	M-009608-00	**TRPM8**	79054	M-006517-01	**YWHAH**	7533	M-010626-01
**TBC1D5**	9779	M-020775-01	**TRPV4**	59341	M-004195-00	**YWHAQ**	10971	M-012329-00
**tcag7.1260**	441282	M-032608-01	**TSPAN6**	7105	M-010624-02	**YWHAZ**	7534	M-003332-01
**TCHP**	84260	M-014843-01	**TSPO**	706	M-009559-03	**ZCCHC17**	51538	M-016869-00
**TECTA**	7007	M-012065-01	**TST**	7263	M-010120-00	**ZNF205**	7755	M-019556-01
**TEGT**	7009	M-004118-01	**TTC19**	54902	M-013361-01	**Bax**	581	M-003308-03
**TF**	7018	M-011189-00	**TTC8**	123016	M-021417-01	**OPA1**	4976	M-005273-00
**TFAP2A**	7020	M-006348-03	**TTR**	7276	M-012554-02			
**TFDP1**	7027	M-003327-04	**TUBA1A**	7846	M-013150-00			
**TFR2**	7036	M-009686-02	**TUFM**	7284	M-016741-00			
**TFRC**	7037	M-003941-02	**TULP1**	7287	M-011413-01			
**TGM2**	7052	M-004971-00	**TUSC2**	11334	M-006478-00			
**THEM4**	117145	M-008604-01	**TXLNA**	200081	M-017959-02			
**TIAM1**	7074	M-003932-02	**TXN2**	25828	M-017448-00			
**TIMM10**	26519	M-008209-01	**TXNRD1**	7296	M-008236-02			
**TIMM13**	26517	M-009538-02	**TYMS**	7298	M-004717-02			
**TIMM17A**	10440	M-012739-01	**U5-200KD**	23020	M-014161-00			
**TIMM17B**	10245	M-020047-00	**UBB**	7314	M-013382-01			
**TIMM22**	29928	M-006473-02	**UBIAD1**	29914	M-020412-01			
**TIMM44**	10469	M-003864-00	**UCP1**	7350	M-007636-01			
**TIMM50**	92609	M-023692-01	**UCP2**	7351	M-005114-00			
**TIMM8A**	1678	M-010342-01	**UCP3**	7352	M-007638-01			
**TIMM8B**	26521	M-008491-00	**UCRC**	29796	M-010899-01			
**TIMM9**	26520	M-009572-00	**ULK1**	8408	M-005049-00			
**TK2**	7084	M-006788-03	**UNC84A**	23353	M-025277-01			
**TMC1**	117531	M-017206-01	**UNQ9438**	387990	M-032055-00			
**TMEFF2**	23671	M-010654-00	**UQCC**	55245	M-017986-00			
**TMEM102**	284114	M-018245-00	**UQCR**	10975	M-032278-00			
**TMEM126A**	84233	M-014829-00	**UQCRB**	7381	M-015787-00			
**TMEM126B**	55863	M-018904-00	**UQCRC1**	7384	M-004748-00			
**TMEM127**	55654	M-020232-01	**UQCRC2**	7385	M-008334-02			
**TMEM14A**	28978	M-018878-01	**UQCRFS1**	7386	M-020100-01			
**TMEM14C**	51522	M-020269-02	**UQCRH**	7388	M-020101-02			

Table S1

**TMEM16F**	196527	M-003867-01	**UQCRQ**	27089	M-012517-01
**TMEM173**	340061	M-024333-00	**USH1C**	10083	M-020028-02
**TMEM192**	201931	M-016802-01	**USH2A**	7399	M-012381-00
**TMEM203**	94107	M-015191-00	**USMG5**	84833	M-014982-01
**TMEM39A**	55254	M-020434-01	**USP30**	84749	M-021294-03
**TMEM39B**	55116	M-021133-00	**USP8**	9101	M-005203-01
**TMEM70**	54968	M-015366-01	**UTRN**	7402	M-012382-01
**TMIE**	259236	M-016265-01	**VAMP1**	6843	M-012497-00
**TMLHE**	55217	M-031958-00	**VAPB**	9217	M-017795-00
**TMPO**	7112	M-027195-02	**VARS2**	57176	M-008268-01
**TNFRSF10B**	8795	M-004448-00	**VAT1**	10493	M-009726-01
**TNFRSF5**	958	M-008101-02	**VAV1**	7409	M-003935-01
**TNFSF10**	8743	M-011524-01	**VCL**	7414	M-009288-01
**TOMM20**	9804	M-006487-00	**VCP**	7415	M-008727-01
**TOMM22**	56993	M-015878-01	**VDAC1**	7416	M-019764-00
**TOMM34**	10953	M-016439-00	**VDAC2**	7417	M-019766-00
**TOMM40**	10452	M-012732-00	**VDAC3**	7419	M-020850-01
**TOMM40L**	84134	M-016907-01	**VEGFB**	7423	M-015731-01
**TOMM7**	54543	M-013762-00	**VHL**	7428	M-003936-00
**TOMM70A**	9868	M-021243-01	**VISA**	57506	M-024237-02
**TP53**	7157	M-003329-03	**VRK2**	7444	M-004684-02

Table S2

siRNAs that increase % **Bax-positive**	siRNAs that increase % **Bax-negative**	siRNAs that increase % **Bax/Omi-positive**
**Plate1**		
MARCH5<br>9.7	EIF3A<br>226.0	BLOC1S1<br>9.5
C19orf12<br>15.3	SLC25A46<br>198.0	C19orf12<br>16.3
BLOCS1<br>16.7	BNIP3<br>196.0	ATCAY<br>23.7
PINK1<br>23.0	DYNLL2<br>195.0	GPER1<br>24.0
MERTK<br>39.0	FMR1<br>192.0	**BNIP3L**<br>30.0
ATCAY<br>43.3	GLTSCR2<br>185.0	MRPL12<br>33.0
SIRT3<br>54.7	RAB11FIP1<br>189.0	IER3<br>37.0
CD68<br>55.3	RAP1B<br>183.0	NR2C2<br>38.7
KLHL7<br>58.3	OPTN<br>170.0	**RMDN3**<br>39.5
STYXL1<br>58.7	MTFP1<br>167.0	ULK1<br>48.3
RNF5<br>65.0	TRAK2<br>155.0	DISC1<br>50.3
NR2C2<br>66.3		FUNDC1<br>59.3
		MARCH5<br>64.0
		LETM1<br>78.8
		RNF5<br>85.3
**Plate 2**		
GNB2L1<br>17.7	PYCR1<br>137.0	TRAK1<br>7.0
SPATA19<br>15.7	AARS<br>136.0	SPATA19<br>15.0
TMEM127<br>23.0	SLC44A1<br>135.0	TRIAP1<br>22.0
ACADL<br>32.7	TIMM50<br>133.7	TMEM127<br>24
PHB<br>37.0	PXN<br>121.7	PHB<br>33.25
FAHD1<br>38.0	SLC25A36<br>110.8	SLC25A14<br>33.3
PLSCR3<br>44.33		**ATG12**<br>42.75
ATG12<br>47.75		
PRELID1<br>56.0		
TRIAP1<br>69.25		
PLA2G6<br>62.25		

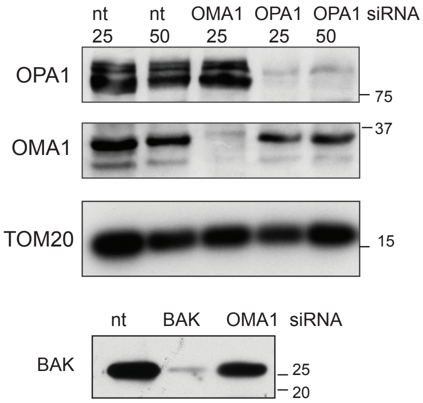
nt 25
nt 50
OMA1 25
OPA1 25
OPA1 50
siRNA
OPA1
75
OMA1
37
TOM20
15
nt
BAK
OMA1
siRNA
BAK
25
20

Figure S1

A

sgRNA	Sequence	Exon	ICE % indels	KO score
**BNIP3L**	GUUCAUGGGUAGCUCCACCC	2	83	74
**RMDN3**	CUCCUGCGGGCUGUCCCAGG	2	94	68
**ATG12**	GUUUCAUACCAACUGUUCUG	2	97	89

B

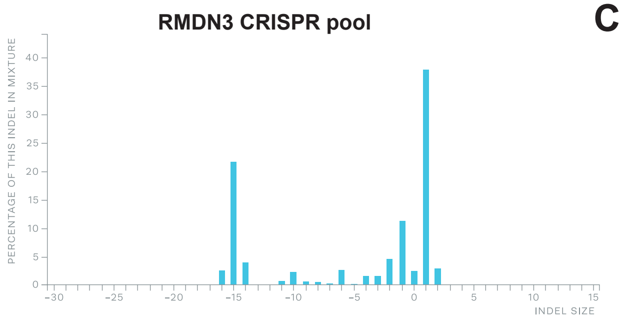

C

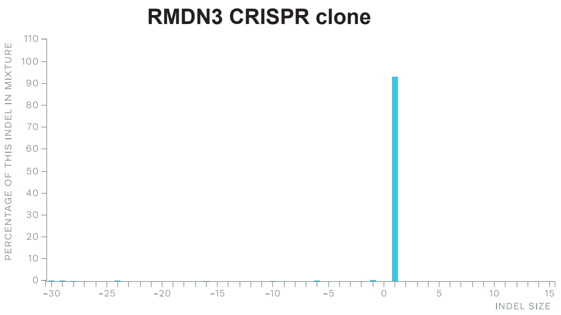

Figure S2
